# Thirty-Fifth Annual Meeting of the Society for Light Treatment and Biological Rhythms (SLTBR), 20 June–22 June, Prague, Czech Republic

**DOI:** 10.3390/clockssleep6040047

**Published:** 2024-11-15

**Authors:** Christian Cajochen

**Affiliations:** Centre for Chronobiology, Research Platform Molecular and Cognitive Neurosciences, Psychiatric Hospital of the University of Basel, Wilhelm-Kleinstr. 27, CH-4002 Basel, Switzerland; christian.cajochen@upk.ch

## 1. Introduction

I am delighted to introduce this collection of abstracts from our recent 35th Annual SLTBR Meeting in Prague. This year’s gathering exemplified the SLTBR’s commitment to advancing knowledge in light treatment and biological rhythms, with a record-breaking number of high-quality submissions, enriching discussions, and collaborative spirit.

Our program spanned a range of key topics, from breakthroughs in circadian medicine and clinical science to innovative translational research. We were honored to have Dr. Satchin Panda as our keynote speaker, whose insights into circadian rhythms and time-restricted eating added a dynamic perspective to our discussions on health and disease. Special thanks go to our board members Corrado Garbazza, Marijke Gordijn, Renske Lok, John Hanifin, Louise Ince, Aleksandar Videnovic, and Lisa Wu for their dedication and collaborative efforts in making this meeting a success.

The Industry Symposium, chaired by Marijke Gordijn and Aleksandar Videnovic, highlighted the invaluable role of our sponsors in shaping the future of circadian health. We are immensely grateful for their support, which makes our work possible and helps bring new perspectives into our field.

I would also like to extend a heartfelt thank you to Annick Goijarts, whose tireless coordination ensured that every detail of our meeting was seamless, as well as to our local hosts, Prof. Zdenka Bendova, and Dr. Lenka Maierova, for their warm hospitality and exceptional organizational efforts in making Prague a perfect setting for our gathering.

Please enjoy reading through these abstracts, which capture the exceptional work and innovative research shared this year. I extend my deepest gratitude to each of you, speakers, organizers, participants, and sponsors alike, for making this meeting a memorable and inspiring experience.

With warm regards,

Christian Cajochen

President, SLTBR

## 2. Conference Abstracts

### 2.1. Cyclin-Dependent Kinase 5 (Cdk5) Activity Is Modulated by Light and Gates Rapid Phase Shifts in the Circadian Clock

Andrea Brenna ^1^, Micaela Borsa ^2^, Gabriella Saro ^1^, Jürgen A. Ripperger ^1^, Dominique A. Glauser ^1^, Zhihong Yang ^1^, Antoine Adamantidis ^2^, Urs Albrecht ^1^

^1^  University of Fribourg, Fribourg, Switzerland
^2^  University of Bern, Bern, Switzerland

**Abstract:** The circadian clock allows organisms to coordinate biochemical and physiological processes over one day. Changes in lighting conditions as they occur naturally over seasons or manmade by jet lag or shift work, advance or delay clock phase to synchronize physiology to the environment. Within the suprachiasmatic nucleus (SCN) of the hypothalamus, circadian timekeeping and resetting have been shown to depend on both membrane depolarization and intracellular second-messenger signaling. In both processes, voltage-gated calcium channels (VGCCs) mediate calcium influx resulting in the activation of intracellular signaling pathways that activate *Period* (*Per*) gene expression. However, the precise mechanism of how these processes are gated in a concerted manner is unknown. Here, we show that cyclin-dependent kinase 5 (Cdk5) activity is modulated by light and gates phase shifts in the circadian clock. We found that knock-down of *Cdk5* in the SCN of mice affects phase delays but not phase advances. This is associated with uncontrolled calcium influx into SCN neurons and an unregulated protein kinase A (PKA)—calcium calmodulin dependent kinase (CaMK)—cAMP response element-binding protein (CREB) signaling pathway. Accordingly, genes such as *Per1* are not induced by light in the SCN of *Cdk5* knock-down mice. Our experiments identified an important light modulated kinase that affects rapid clock phase adaptation. This finding indicates how light responsiveness and clock phase are coordinated to adapt activity onset to seasonal changes, jet lag, and shift work.

### 2.2. Evaluation of a Behavioral Intervention in a Municipal Context (“Light, Activity and Sleep in My Daily Life”): A Pilot Study Protocol

Kiran Maini Gerhardsson, Christina Brogårdh, Åsa B Tornberg, Steven M. Schmidt

Lund University, Lund, Sweden

**Abstract:Background**: While it is well established that having the appropriate electric lighting, exposure to daylight, opportunities for physical activity, and good sleep are interrelated, health-promoting strategies directed at older adults have so far never targeted both behavior and environmental proactivity. A behavioral intervention (‘Light, activity and sleep in my daily life’) was developed to address this need. The intervention aims to support changes to routines (physical activity outdoors and sleep routines) and in the home (light-related adjustments). It is delivered as a web-based course consisting of nine weekly online modules, three to four physical meetings held at the senior citizen meeting place and a test kit to encourage experimentation in the home. Usability testing was conducted in a lab and the field, enabling content refinement. This longitudinal pilot study (2024–2026) aims to evaluate the intervention’s usability, acceptance and effectiveness in four municipalities in Sweden. Guided by health-related behavioral change theories, the intervention could lead to the desired changes and be sustained over time, assuming the intervention participants have the capability (i.e., have the physical and cognitive capacity), are motivated (i.e., find the course to be enjoyable and relevant to their needs), and have the opportunity (i.e., a supportive learning environment, the technical infrastructure at home, and a walk-friendly outdoor environment). **Methods:** A case study design will be used because the municipality-implemented intervention (the case) heavily depends on the specific setting (the context), e.g., the urban outdoor environment and available senior citizen meeting points. Another reason for adopting a case study design is the need for an in-depth understanding of both micro (individual) and meso (service provision) levels. Multiple kinds of data will be obtained from (1) community-dwelling individuals (≥70 yrs) participating in the intervention to assess its usability and intervention outcomes (sleep, physical activity, core affect, quality of life, self-efficacy, computer anxiety, self-managed changes in the home) and (2) employees in the municipal healthcare service participating in focus groups to assess their acceptance of intervention delivery procedures and future implementation. Health-related intervention outcomes will be collected before and directly after the intervention and after three and ten months, including data from wrist-worn accelerometers, questionnaires, interviews, and observations (light-related adjustments in the home and outdoor characteristics in the urban environment). **Expected Results**: Potential immediate benefits include maintaining the intervention participants’ good health by increasing their control over daily routines and home lighting conditions and developing their digital skills. Potential intermediate benefits are fewer fall accidents at home because of better lighting conditions during the day and night and readiness to use e-health applications. Potential ultimate benefits include continued independent living in the community, civic participation, and improved quality of life. If the intended outcomes are met and implementation procedures are feasible, such an intervention can benefit the older population’s health behavior and society. **Conclusions**: The pilot will enable us to trial the study approach and refine procedures before a more extensive future case study.

**Keywords:** intervention; behavior; quality of life; older adults

**Funding:** The Kamprad Family Foundation.

### 2.3. Reproducible Analysis of Personal Light Exposure Data with LightLogR

Johannes Zauner ^1^, Steffen Hartmeyer ^2^, Manuel Spitschan ^1^

^1^  Technical University of Munich, Munich, Germany
^2^  EPFL, Lausanne, Switzerland

**Abstract:Background**: Personal light exposure is associated with many health outcomes. Conventionally, light is characterized using spot measurements with stationary equipment. In field studies, where (near-)corneal measurements from wearable light loggers and dosimeters are the best proxies to link personal light exposure to human responses, spot measurements are generally not possible or practical. However, time-series light exposure data are considerably more complex than singular spot measurements. The research field currently lacks a common and unified framework for collecting, processing, analyzing, and sharing these data FAIRly (i.e., in findable, accessible, interoperable, and reusable ways). **Methods**: Within the EURAMET-funded project MeLiDos (metrology for wearable light loggers and optical radiation dosimeters) and the CIE Joint Technical Committee 20 (D2/D6), we worked on developing common standards for device characterization and calibration, developing the best practices in data collection, processing, and analysis, including a software ecosystem and identifying and probing future directions for spatially resolved light logging. A community-driven and consensus-based approach was integral to all these efforts. All software development was open source, and collaborators, testers, and interested parties were actively invited. The first output was an R software package to facilitate valid and reproducible processing and analysis of light measurements from wearable devices. **Results**: We have developed LightLogR, an R software package for working with personal light exposure data from wearable light loggers and dosimeters. LightLogR contains the following functionality: import routines for data from many common wearable devices; validation and processing functions to find and handle gaps in the data, aggregate data, or deal with daylight savings time; augmentation with auxiliary data (e.g., sleep-diary, wear log, questionnaire data); powerful visualization functions for exploratory insights and publication-ready graphics; light exposure metrics: intra-daily variability, inter-daily stability, time above threshold, brightest/darkest hours of the day, and many more; and analysis routines for confirming compliance of actual light exposure to experimental procedure or other thresholds. Robustness and accessibility of the package were facilitated through a rigorous open-source approach (GPL-3.0 license; source code https://github.com/tscnlab/LightLogR, accessed on 30 May 2024), unit testing, and in-depth documentation (https://tscnlab.github.io/LightLogR/, accessed on 30 May 2024). **Conclusions**: LightLogR is a standard pipeline for analysis and is already usable today as part of a larger effort to make research collecting wearable light data more valid and impactful. The package is under active development with a small community of research users worldwide. The roadmap until 2026 contains several features, including a Shiny implementation for a GUI-based web platform of the LightLogR functionality, semi-automated non-wear detection, the generation of data/metadata files, and validation of such files for FAIR data, the integration of data into a unified database for cross-study analyses, and semi-automated analyses and visualization.

**Keywords**: personal light exposure; light loggers; dosimetry; wearables; FAIR; analysis; R statistical software; open source

### 2.4. How to Measure, Characterize, and Report Light in Studies and Clinical Trials

Manuel Spitschan

Technical University of Munich, München, Germany

**Abstract:** Light can be described and parametrized through various parameters, including intensity, spectral composition, wavelength, duration, timing, and spatial configuration. In studies and clinical trials using light as an intervention, it can be non-trivial to know what to measure and report. In this introduction, I will provide a framework for measuring, characterizing, and reporting light based on the ENLIGHT Checklist.

### 2.5. Sensitivity of the Circadian System to Light in Early Childhood

Lauren Hartstein ^1^, Kenneth Wright ^2^, Cecilia Diniz Behn ^3^, Shelby Stowe ^3^, Monique LeBourgeois ^2^

^1^  University of Arizona, Tucson, AZ, USA
^2^  University of Colorado Boulder, Boulder, CO, USA
^3^  Colorado School of Mines, Golden, CO, USA

**Abstract:Background**: The extensive literature details the effects of light exposure on the circadian clock in adult populations. Little is known, however, about the sensitivity of the circadian system to the characteristics of light (intensity, spectrum, timing) in early childhood, a pivotal phase in the maturation and consolidation of sleep and a time when sleep problems often first emerge. **Methods**: Across 3 separate studies, healthy preschool-aged children (3.0–5.9 years) completed a 3-day in-home dim-light circadian assessment in which they were exposed to light for 1 h in the morning or evening, differing in either spectrum or intensity. Salivary melatonin levels were assessed to determine acute suppression of melatonin during evening light exposures as well as changes in circadian timing (DLMO) as a result of each light condition. **Results**: Children demonstrated high sensitivity to light in the hour before bedtime, with robust melatonin suppression and phase delays observed across a wide range of light intensities (5–5000 lux). Evening exposure to light of a higher CCT (5000 K) caused greater suppression of melatonin than light of a lower CCT (2700 K) at only 20 lux, but both resulted in significant delays of the circadian clock. Conversely, children’s circadian timing was minimally impacted by light presented in the hour after waking (1.5–2000 lux). **Conclusions**: Together, these findings point to evening light as a modifiable factor in children’s environments that could be contributing to the development of behavioral sleep problems through impacts on children’s circadian physiology. Further work is vital in order to understand photosensitivity across child development and inform recommendations for parents on light exposure profiles to support healthy sleep.

**Keywords**: children; light; circadian rhythms; melatonin

**Funding:** Eunice Kennedy Shriver National Institute of Child Health & Human Development (F32-HD103390; R01-HD087707) and the National Heart, Lung, And Blood Institute of the National Institutes of Health (T32-HL149646).

### 2.6. Bedroom Light Exposure at Night and Obesity in Individuals with Bipolar Disorder

Rina Taniguchi ^1^, Yuichi Esaki ^1^, Keigo Saeki ^2^, Kiyoshi Fujita ^1^, Kenji Obayashi ^2^, Tsuyoshi Kitajima ^3^, Nakao Iwata-Japan ^3^

^1^  Department of Psychiatry, Okehazama Hospital, Toyoake, Aichi, Japan
^2^  Department of Epidemiology, Nara Medical University School of Medicine, Nara, Japan
^3^  Department of Psychiatry, Fujita Health University School of Medicine, Toyoake, Japan

**Abstract:Background**: Obesity and overweight are highly prevalent in individuals with bipolar disorder (BD) and are associated with a risk of developing not only physical but also mental problems. A recent study showed that light at night (LAN) exposure was significantly associated with circadian disruption and poor sleep quality in BD patients. However, no study has investigated the association between LAN and obesity in individuals with BD. This cross-sectional study was to determine the relation between bedroom light exposure at night and obesity in BD. **Methods:** Two hundred participants were identified from the outpatients with BD who participated in a study titled “Association between the Pathology of Bipolar Disorder and Light Exposure in Daily Life (APPLE) cohort study” between August 2017 and October 2019. They were instructed to record bedroom light from bedtime to rising time using a portable photometer, and LAN exposure in the bedroom was recorded at 1 min intervals. Body mass index (BMI) was determined using self-reported height and weight, and obesity was defined as a BMI ≥ 25 kg/m^2^. **Result**: The mean (standard deviation) weight, height, and BMI were 66.3 (15.9) kg, 1.63 (0.09) meter, and 24.8 (5.2) kg/m^2^, and 88 (44%) patients were obese. The median (interquartile range) of the average LAN was 2.2(0.3–10.2) lux, and 88 (44.0%) participants were exposed to an average LAN ≥ 3 lux. The odds ratio (OR) for obesity was significantly higher in the group exposed to an average LAN ≥ 3 lux than in the group exposed to an average LAN < 3 lux (OR:2.16, 95% confidence interval: 1.22–3.82). In the multivariable logistic regression analysis, after adjusting for several confounding factors like age, gender, and medications, a similar result was observed (OR: 2.23, 95% confidence interval:1.19–4.21). Furthermore, the patients with an average LAN ≥ 3 lux had significantly higher body weight (adjusted mean, 68.7 vs. 64.4 kg; *p* = 0.033) and BMI (25.6 vs. 24.2 kg/m^2^; *p* = 0.046) than those with an average LAN < 3 lux. **Conclusions**: Significant association between bedroom light exposure at night and obesity was observed. LAN exposure was also significantly associated with continuous data on body weight and BMI, independent of confounding factors. Further longitudinal investigations are necessary to validate the association between LAN exposure and obesity in BD.

### 2.7. Can Time-Restricted Feeding Prevent the Disturbing Behavioral Consequences of Dim Artificial Light at Night?

Valentína Sophia Rumanová ^1^, Ewout Foppen ^2^, Michal Zeman ^3^, Monika Okuliarova ^3^, Andries Kalsbeek ^1^

^1^  Department of Endocrinology and Metabolism, Amsterdam UMC, University of Amsterdam, Amsterdam, The Netherlands
^2^  Laboratory of Endocrinology, Amsterdam University Medical Centers, Amsterdam Gastroenterology Endocrinology Metabolism (AGEM), Amsterdam, The Netherlands
^3^  Department of Animal Physiology and Ethology, Faculty of Natural Sciences, Comenius University, Bratislava, Slovakia

**Abstract:** To stay synchronized with the rhythmic changes in the environment, organisms developed an internal timekeeping system that enables them to predict these changes and adapt their physiology accordingly. External cues, such as the light-dark (L/D) cycle, synchronize these biological rhythms (also known as circadian rhythms) via outputs from the central oscillator in the hypothalamic suprachiasmatic nuclei. Disrupting the natural L/D regime can cause the internal desynchronization of the circadian system, resulting in metabolic and cardiovascular health issues. One of the modern-day disruptors of the internal clock is exposure to artificial light at night (ALAN). Over the last few decades, light pollution has been rising due to the excessive use of streetlights. An increase in the light illumination outside of our homes not only harms the environment and many ecosystems, but also exposes people to low-intensity light in their homes. Many epidemiological studies associate ALAN with cardiometabolic diseases, mental health issues and sleep disorders. However, the underlying mechanisms still need to be clearly understood. Our previous studies showed that dim ALAN disrupts the daily rhythms of the central clock, its outputs, and the timing of metabolic pathways and the clockwork mechanism in peripheral tissues. Behavioral measurements revealed reduced daily variability in locomotor activity, and food and water intake, with an increase during the rest period and reduction during the active period. One of the most surprising findings was reduced water intake during the final 2 h of the night when animals usually drink extra in anticipation of the rest period. In this project, we used a time-restricted feeding protocol to investigate if these ALAN-induced behavioral changes can be prevented, as it is well-known that restricting food intake to only the active period helps to strengthen daily rhythms and improve the metabolic flexibility of an organism. Male Wistar rats were kept in a 12/12 = L/D cycle and food was available ad libitum. After a week of adaptation to the metabolic cages, we divided the animals into two groups, with ad libitum food access for 24 h/day or time-restricted food during the 12 h dark period (TRF-n) for one week. This was followed by a two-week exposure to dim ALAN (~2 lx) throughout the night, while at the same time maintaining the ad libitum and time-restricted conditions. As expected, TRF-n elevated the amplitude of the food intake rhythm. However, ALAN still suppressed the rhythm of locomotor activity, with an additional peak during the rest period and an eliminated bimodal pattern during the night despite food restriction. Lastly, TRF-n did not prevent the drop in anticipatory drinking and ALAN still suppressed the daily variation in water intake. In conclusion, we found that TRF-n strengthens the daily feeding rhythm, but it did not prevent the ALAN-induced changes in locomotor activity and water intake. Thus, ALAN still resulted in a misalignment between these behavioral outputs. Our findings underscore the importance of an undisturbed L/D cycle for the proper timing of behavioral rhythms.

**Funding:** This research was funded by ZonMw Open Competition grant #09120012010051, The Slovak Research and Development Agency #APVV-21-0223 and by The Scientific Grant Agency of the Ministry of Education, Science, Research and Sport #VEGA-1/0309/23.

### 2.8. Monochromatic and Polychromatic Light Suppression of Nocturnal Melatonin in Adult Men Stimulates Prostate Cancer Growth and Metabolism in Human Prostate Cancer Xenografts in Rats

George Brainard ^1^, Robert Dauchy ^2^, John Hanifin ^1^, Erin Dauchy ^2^, Benjamin Warfield ^1^, Kate Cecil ^1^, Victoria Belancio ^2^, Lulu Mao ^2^, Melissa Wren-Dail ^2^, Shulin Xiang ^2^, Steven Hill ^2^, David Blask ^2^

^1^  Thomas Jefferson University, Philadelphia, PA, USA
^2^  Tulane University, New Orleans, LA, USA

**Abstract:Background**: Light has the capacity to restore human health in clinical applications such as treating winter depression and selected sleep disorders. Any agent that has the capacity to heal, however, also has the capacity to harm. In 1987, it was hypothesized that the increased risk of breast cancer in industrialized countries is due, in part, to increased exposure to electrical light at night that suppresses melatonin secretion from the pineal gland (Stevens, 1987). Since then, epidemiological studies have supported the “light, melatonin, breast cancer hypothesis” (e.g., Schernhammer et al., 2001; Stevens et al., 2014). Empirical evidence from both in vivo and in vitro animal studies also supports this hypothesis. Importantly, it has been demonstrated that human breast cancer xenografts perfused in situ with nocturnal, physiologically melatonin-rich blood collected from premenopausal female volunteers during the night exhibited suppressed breast cancer proliferation. This finding identified a definitive nexus between the exposure of healthy premenopausal female human subjects to light at night and the enhancement of human breast cancer development via disruption of the circadian melatonin signal (Blask et al., 2005). The following two studies tested the hypothesis that polychromatic and monochromatic light exposure at night would suppress melatonin in men and lead to human prostate tumor growth and metabolism. **Methods**: In Study 1, blood samples were collected from healthy human male volunteers. Samples were obtained during the daytime hours, nighttime hours (2:00 AM) in darkness, and nighttime following 90 min of full field bright fluorescent light exposure from 2:00 to 3:30 AM of 1853 melanopic Equivalent Daytime Illuminance (melanopic EDI, or 2800 photopic lux, per CIE S 026, 2018). In Study 2, samples were collected from a different set of men during daytime hours, nighttime hours (2:00 AM) in darkness, nighttime following 90 min of full field monochromatic light exposure of equal photon flux (9.8E + 15 cm^2^/sec) of monochromatic light at 480 or 630 nm (289 or 0.1 melanopic EDI, or 39 or 58 photopic lux, respectively), and a dark control condition. Human blood samples from each study were perfused into tissue isolated, PC3 human prostate tumor xenografts in nude rats. Human blood levels of melatonin were quantified, and tumor progression and metabolism were assessed. **Results**: Perfusion results showed significant reductions (*p* < 0.05 to *p* < 0.001) in prostate tumor cAMP levels, total fatty acid and linoleic acid uptake, 13-HODE production, glucose uptake, and [^3^H] thymidine incorporation into tumor DNA for melatonin-rich dark control nights (Studies 1 and 2) and nighttime/630 nm light exposure (Study 2). In contrast, the human prostate tumors had significantly increased growth and metabolism when perfused with melatonin-diminished human blood after exposure to bright polychromatic light (Study 1), 480 nm monochromatic light (Study 2) and daylight (Studies 1 and 2). **Conclusions**: These results show that nighttime light exposures that suppress normally high levels of melatonin in human blood can stimulate human prostate tumor growth and metabolism.

**Keywords**: polychromatic light; monochromatic light; melatonin; prostate cancer

**Funding:** Supported by the Nova Institute for Health (formerly TIIH), Tulane Cancer Center, and Louisiana Cancer Research Consortium.

### 2.9. Early Life Exposure to Blue Light at Night in the Diurnal Rodent Arvicanthis ansorgei Affects the Circadian System in Adulthood

Axelle Date ^1^, Marie-Paule Felder-Schmittbuhl ^1^, Ouria Dkhissi-Benyahya ^2^, Jorge Mendoza-France ^1^

^1^  University of Strasbourg, Alsace, France
^2^  University of Lyon, Lyon, France

**Abstract:Background**: Nowadays, light-emitting diodes (LEDs), with greater emission in short wavelengths (e.g., blue light), are omnipresent in our daily lighting sources (indoors and outdoors) as well as in electronic devices (e.g., televisions, smartphones, etc.) and used by a large part of the population. Adolescents are part of the population particularly vulnerable to the lighting environment at night because they present a late chronotype and (due to differences in pupil and lens) increased sensitivity to evening light. According to the National Sleep Foundation, about 60% of teens report interacting with screens in the hour before bedtime, compromising sleep and circadian rhythms. Aberrant exposure to light (ALE) at young ages (adolescence) could have negative consequences in health, persisting even in adulthood, but this has not been fully explored. Numerous studies using nocturnal adult rodents show that the chronic use of blue light at night (BLAN) can lead to alterations in many physiological functions, and, in particular, to the disruption of circadian rhythms and sleep. In the present study, in order to understand how BLAN exposure might affect human physiology, we evaluated the impact of BLAN in early life on the circadian system in adulthood, using the diurnal rodent *Arvicanthis ansorgei*. **Methods**: We used three groups of animals exposed to different lighting conditions at the juvenile (“adolescent”) period (P20-P40): 1: a control group (Cont) exposed to a 12L/12D light-dark (LD) cycle (white light; 480–800 nm, 275 lux; 2), a blue light group (BLAN) exposed to 3 h of blue light (480 nm, 500 lux; 10^14^ photons/cm2/s; 3), and a white light group (WLAN), exposed to 3 h of white light (440–800 nm, 275 lux). In both the BLAN and WLAN groups, 3 h of light exposure occurred at the beginning of night (ZT12-ZT15; ZT0 = lights on). In order to evaluate the effects of ALE on the circadian system at adulthood (P60), locomotor activity rhythms were analyzed (e.g., onset and offset, period, activity profile, etc.). Finally, under constant darkness conditions, phase shifts in response to acute (15 min) monochromatic light stimulation (480 nm) at CT14 were assessed. **Results**: We showed that BLAN-exposed animals present a delay in the offset of daily rhythms of activity, maintained in adulthood (p60). The average activity of these animals during the ZT12-ZT15 period remains significantly higher in adulthood compared with animals in the WLANgroup. Finally, our results indicate that in response to a blue light pulse at CT14, BLAN-exposed animals present a significantly larger phase delay of locomotor activity rhythm (around 1 h), compared with animals in the Cont and WLAN groups (around 30 min). **Conclusions**: These results suggest that BLAN exposure at early life induces an alteration of the circadian system, which persists in the long term (adulthood). The characterization of the underlying neuroanatomical mechanisms will be crucial for a better understanding of the effects of BLAN in physiology and behavior.

### 2.10. Time-of-Day-Dependent Metabolic Adaptations to Endotoxin Are Compromised by Artificial Light at Night

Monika Okuliarova-Slovakia, Beata Benedikova, Viera Jerigova, Michal Zeman-Slovakia

Department of Animal Physiology and Ethology, Faculty of Natural Sciences, Comenius University, Bratislava, Slovakia

**Abstract:Background**: Immune defense is a highly energy-demanding process that requires systemic metabolic changes to redirect energy and nutrients, such as glucose and fatty acids (FA), to activated immune cells. The circadian system governs the daily rhythms in immune and metabolic parameters and synchronize them with external environmental cycles. Accordingly, the nature of the immune response varies throughout the day but the temporal dependence of metabolic adaptations following immune challenge is poorly understood. A further question is whether metabolic changes during inflammation are sensitive to the disruption of the light-dark (L/D) cycle, which is a prominent Zeitgeber for the circadian clock. Previously, we showed that dim artificial light at night (ALAN) attenuated the anorectic response to endotoxin when rats were challenged during their early light phase and suppressed the daily variability of the inflammatory response in blood leukocyte counts. In the current study, we examined whether lipopolysaccharide (LPS)-induced responses in glucose and lipid metabolism depend on the time of exposure and to what extent they can be affected by ALAN. **Methods**: Adult male Wistar rats were exposed either to a standard 12/12 h L/D cycle or ALAN (~2 lx) during the whole dark phase for 2 weeks. Thereafter, rats were challenged with LPS at the early light (ZT2) or the early dark/dim light (ZT14) phase and acute immune and metabolic changes were analyzed in the plasma, liver, and visceral white adipose tissue (WAT). **Results**: Time-of-day-dependent response to LPS was observed for hepatic glucose and FA transporters and FA synthase (FASN). Specifically, daytime LPS upregulated *Glut1*, whereas nighttime LPS upregulated *Cd36* and downregulated *Fasn* expression with no changes in the opposite phase. ALAN suppressed the *Cd36* response and induced downregulation of hepatic sirtuin 1 (*Sirt1*) after nighttime LPS. In the WAT, the expression of *Glut4* and carnitine palmitoyltransferase 1B decreased only after daytime LPS challenge, whereas the expression of nicotinamide phosphoribosyltransferase (*Nampt*) increased. ALAN reduced the LPS-induced daytime upregulation of *Nampt*. Furthermore, plasma leptin and adiponectin decreased after daytime LPS injection in LD rats, whereas no response was found in ALAN rats. The suppressed metabolic response to daytime LPS under the ALAN regime was associated with suppressed immune response in the WAT. On the other hand, ALAN rats showed an upregulation of adipose ATP-citrate lyase and a trend towards upregulation of *Glut4* upon nighttime LPS. Interestingly, liver and adipose peripheral clocks only responded to nighttime LPS challenge. ALAN inversely changed the clock gene expression in the light and night phase and diminished LPS-induced upregulation of *Bmal1* and *Nfil3* in the liver. **Conclusions**: The metabolic response to LPS, particularly in genes involved in glucose and FA uptake and utilization, showed the time-of-day-dependent variation and was altered by disruptions of the metabolic clocks by ALAN.

**Funding:** Supported by APVV-21-0223 and VEGA 1/0565/22.

### 2.11. Investigating the Impact of Light Exposure on Mood in Everyday Life: A Naturalistic Study

Chloe Roddis ^1^, Altug Didikoglu ^2^, Amy Gillespie ^3^, Catherine Harmer ^3^, Beatriz Bano Otalora ^1^, Nina Milosavljevic ^1^

^1^  Centre for Biological Timing, Division of Neuroscience, School of Biological Sciences, Faculty of Biology Medicine and Health, University of Manchester, Manchester M13 9PL, UK
^2^  Department of Neuroscience, Izmir Institute of Technology, Gulbahce, Izmir 35430, Turkey
^3^  Department of Psychiatry, University of Oxford, Oxford OX3 7JX, UK

**Abstract:** In the last decade, it has become apparent that light is not only important for vision, but that light responsiveness extends to fundamental aspects of mood, cognition, and performance. Light can be used as an effective and non-invasive therapeutic option with little to no side effects to improve sleep, mood, and general well-being. However, to utilize the full potential of light as therapy and design new interventions, further research is required, starting with determining the relationship between light and mood in human subjects in real-life situations. This study’s methodology will be the first to offer information on naturalistic light exposure situations and provide insight into its relationship with emotional bias in participants. In total, 25 healthy volunteers were recruited for a 1-week light monitor study. Participants received two devices—a light monitor (which measures the gold-standard unit of ambient light exposure, Melanopic-EDI) for daytime use and a Fitbit for continuous sleep and physiology tracking (activity and sleep patterns, heart rate). They were given access to an online questionnaire link. During the week, participants were asked to complete questionnaires, including a baseline questionnaire, which asked for information on sleep health, general health, and demographics, in addition to a daily sleep diary and tasks aimed at assessing their current mood status. This included both repeat subjective mood questionnaires and daily objective cognitive tests of emotional bias, based on using validated tests including the Emotional Categorization Test (ECAT), Emotional Recall Task (EREC), and Emotional Recognition Memory Task (EMEM). This was also the first time these objective mood questionnaires were used across the week, and they were adapted to be conducted on separate days, not just in one sitting. Here, we describe the preliminary results of five healthy volunteers, which demonstrated the feasibility of the measurements we are collecting. We gathered, on average, 7.5 days of continuous melanopic EDI data from everyday life, proving the acceptability of our light monitoring protocol. Preliminary results suggested there was no significant difference between days for word recall. There was also a non-significant correlation between the objective positive bias scores (ECAT/EREC/EMEM) and subjective mood scores. However, as mood varied throughout the day, analyzing subjective questionnaires submitted at the same time of day as objective questionnaires was beneficial. Future work will focus on analyzing circadian and light exposure impacts on mood regulation. Collectively, these data should reveal the daily rhythmicity of mood, the efficacy of objective emotion tasks in detecting mood variations in real-world settings, and the relationship between light exposure, physiological variables, and mood in healthy volunteers.

### 2.12. Solid State Lighting Countermeasures to Improve Color Vision and Melatonin Onset During a High-Fidelity Analog Study for the International Space Station (ISS)

John Hanifin ^1^, Mijail Serruya ^1^, Benjamin Warfield ^1^, John Kemp ^1^, Taehwan Yoo ^1^, Melissa Ayers ^1^, Robert Soler ^2^, Steven Lockley ^3^, George Brainard ^1^,

^1^  Thomas Jefferson University, Philadelphia, PA, USA
^2^  Biological Innovation and Optimization Systems, LLC., Carlsbad, CA, USA
^3^  Harvard Medical School, Boston, MA, USA

**Abstract:Background**: On space shuttle and ISS missions, crewmembers obtained about one hour less sleep on nights when their circadian rhythms were misaligned [1,2]. Light can be a powerful countermeasure for both circadian misalignment and sleepiness. The ISS was originally equipped with fluorescent General Luminaire Assemblies (GLAs) for illuminating the astronauts’ working and living environments. The GLAs have been replaced in stages with Solid State Lighting Assemblies (SSLAs) that emit three predefined color temperature settings [3]. To date, 84 SSLAs have replaced 99% of the original GLAs. The aim of the following work was to evaluate light emitted by SSLAs for efficacy in supporting astronaut operational tasks and regulating neuroendocrine physiology. **Methods**: A dynamic lighting schedule was developed based on the spectral and intensity sensitivities of the human circadian photoreceptor system. The SSLAs have three light settings, each with a unique intensity and spectrum to optimize their efficacy: (1) General Vision; (2) Alertness/Phase Shift; and (3) Pre-Sleep. Proper uses of the SSLAs are intended to (1) facilitate circadian adaptation; (2) enhance sleep; and (3) improve alertness and performance, while maintaining high visual acuity and color discernment for operational tasks. Previously, three analog studies were conducted with healthy astronaut-aged volunteers: a 5-day, controlled in-patient study to test the efficacy of a lighting protocol for daily operations that utilizes SSLAs and two studies on color vision and visual performance. Those studies were conducted in the high-fidelity ISS analog crew quarters laboratory at Thomas Jefferson University (TJU). In addition, an in-flight study with seven astronauts at the ISS was successfully completed. Data from those studies led to the current analog study assessing a modification of the SSLA Pre-Sleep setting. Eight healthy, astronaut-aged men and women participated in a randomized, three condition study involving SSLA exposures of General Vision, the original Pre-Sleep setting, and a new Pre-Sleep setting that was modified in light irradiance and wavelength. These light exposures were given in a high-fidelity replica of an ISS crew sleeping quarters at TJU. Dependent variables included dim light melatonin onset (DLMO), a Farnsworth–Munsell 100 (FM100) color vision test, and a Lanthony desaturated D15 color vision test. **Results**: Eight subjects completed all the study nights. FM100 color vision testing results revealed statistically significant differences (N = 8, F = 4.84, *p* < 0.05) in color vision discrimination scores relative to the different light settings of the SSLAs. Preliminary assessment (N = 4) of DLMO showed a delayed onset of plasma melatonin levels under the General Vision setting compared with each Pre-Sleep setting. Further data analysis is ongoing. **Conclusions**: Risk factors for the health and safety of astronauts include disturbed circadian rhythms and altered sleep–wake patterns. This study’s results will determine if SSLA lighting can be used to support astronaut vision as well as serve as an in-flight countermeasure for circadian misalignment, sleep disruption, and performance deficits at the ISS. Appropriately designed lighting systems will serve as a countermeasure to mitigate such risks in the newer NASA space exploration programs such as Artemis and Gateway.

**Keywords**: light, sleep, color vison, melatonin, spaceflight.

**Funding:** Primary funding: NASA #NNX15AC14G. Additional support: NASA #NNX09AM68G, NSBRI through NASA NCC 9-58, The Nova Institute for Health (formerly TIIH), and the Philadelphia Section of the IESNA.

**References**

1. Barger et al. (2014) *Lancet Neurol* 13, 904–912.
2. Flynn-Evans et al. (2016) *NPG Micrograv* 2, 1–6.
3. NASA Specification (2013) S684-13489.

### 2.13. Dose–Response Relationship of Light-Induced Melatonin Suppression in East Asians

Yusuke Nakazawa ^1^, Kazuki Imaizumi ^1^, Taisuke Eto ^2^, Toshiyuki Hayakawa ^3^, Shigekazu Higuchi ^1^

^1^  Human Life Design and Science Course, Graduate School of Design, Kyushu University, Fukuoka, Japan
^2^  Department of Sleep–Wake Disorders, National Institute of Mental Health, National Center of Neurology and Psychiatry, Tokyo, Japan.
^3^  Faculty of Arts and Science, Kyushu University, Fukuoka, Japan

**Abstract:Background**: There is growing interest in research on individual differences in sensitivity to the non-visual effects of light. In the present study, we investigated the dose–response relationship of light-induced melatonin suppression in East Asians and compared the results with those of studies in Western countries to examine ethnic differences in light sensitivity. **Methods**: Seventeen healthy East Asian university students (22.4 ± 1.53 years/9 males) with no eye disease or color blindness participated in the experiment. Participants were exposed to each light condition (Dim, 30, 100, 400, and 2000 lx) for 3 h after 2.5 h exposure to dim light (<3 lx). The experiment was conducted in a crossover design, and the order of light exposure was counterbalanced. Light exposure started approximately one hour after the DLMO (dim light melatonin onset), measured before the experiment. The percentage of light-induced suppression of salivary melatonin concentration was used as an index of light sensitivity. The predictive model of the melatonin suppression rate of Giménez et al. (2022) was used as comparative data for European ethnic groups. **Results**: A significant main effect of illuminance on the percentage of melatonin suppression was found in East Asian people (*p* < 0.001), and there was a clear dose-dependent relationship between illuminance and suppression of melatonin. Next, we converted each illuminance level in this experiment to mEDI (melanopic equivalent daylight illuminance) and calculated the percentage of melatonin suppression from the predictive model of Giménez et al. (2022). The results showed that melatonin suppression in Asians was smaller than that predicted by Giménez’s model at low levels of light with mEDI around 30–100 lx. **Conclusions**: The present study suggests that ethnic differences exist in light-induced melatonin suppression, and East Asians may be less sensitive than European ethnic groups.

### 2.14. Reliability of Field-Measured Timing of Melatonin Onset with Minimal Instructions and Self-Reported Sleep Timing

Larissa Wüst, Christian Cajochen, Ruta Lasauskaite

Centre for Chronobiology, Psychiatric Hospital of the University of Basel, Basel, Switzerland and Research Cluster Cognitive and Molecular Neurosciences (MCN), University of Basel, Basel, Switzerland

**Abstract:Background**: Research suggests that alertness and task performance depend on the optimal or non-optimal time regarding an individual’s chronotype. Chronotypes are often determined from questionnaires and are associated with the dim light melatonin onset. In an exploratory analysis, we assessed associations between self-reported sleep timing and timing of melatonin onset from saliva samples collected at home with minimal instructions. Further, we assessed associations of chronotypes derived from sleep time and timing of melatonin onset on alertness, perceived task difficulty, and task performance in a cognitive challenge presented in the morning. **Methods:** Forty participants (18–34 years, 24 women) underwent an experimental protocol of two times 5 nights on a fixed sleep–wake schedule, adapted to their habitual sleep timing, and an experimental session in the laboratory. The sleep–wake schedule included an 8 h sleep opportunity for the first 4 nights and either 8 h or 5 h sleep opportunity in the fifth night, in a counterbalanced order. On this evening, participants independently collected hourly saliva samples, starting 5 h prior to their habitual sleep time. Participants were only instructed to refrain from caffeine and alcohol during saliva collection. Melatonin concentration was analyzed using RIA assays and timing of melatonin onset was determined using the hockey stick method (Danilenko et al., 2014). Timing of melatonin onset was averaged from both assessments of each participant. During the experimental sessions, scheduled 2 h after habitual wake-up, a 5 min auditory 2-back task was completed. Participants rated their alertness on the Karolinska Sleepiness Scale (Akerstedt and Gillberg, 1990) and task difficulty on a 7-point scale. Sleep times were assessed using the Munich Chronotype Questionnaire (MCTQ, Roenneberg et al., 2003). Reported sleep times for workdays and free days were averaged for habitual sleep time. Chronotypes were categorized from averaged sleep time and from averaged timing of melatonin onset by splitting the dataset into thirds (early, intermediate, and late) based on each of the measures. **Results:** Timing of melatonin onset from at least one sleep condition was available for 39 participants, and valid MCTQ data were provided by 38 participants. We found a strong correlation of the averaged sleep time and averaged timing of melatonin onset (r = 0.733, *p* < 0.001). Chronotype categorizations, based on the MCTQ and on melatonin onset, showed low consistency (*Cronbach’s alpha* = 0.578). No associations of sleep time-derived chronotypes with alertness, perceived task difficulty, and task performance were found (*p*s > 0.067). Task performance was better in early melatonin-derived chronotypes (*F_2,35.27_* = 4.46, *p* = 0.019), but no differences in alertness or perceived difficulty were found (*p*s > 0.591). **Conclusions:** Our exploratory analysis indicates that even with minimal instructions to participants, which did not include exposure to light, it is possible to reliably estimate the timing of melatonin onset. This is an important finding for future field studies but also for possible clinical applications. Furthermore, our data suggest that the association of chronotypes with alertness, task performance, and perceived task difficulty in the morning appears to be marginal, when the time of assessment is adapted to habitual wake-up.

**Keywords**: sleep; melatonin; chronotype; alertness; performance

**Funding:** This work was funded through the SNSF grant PZ00P1_179953 awarded to RL.

### 2.15. Measuring, Detecting, and Handling Non-Wear Intervals in Longitudinal Light Exposure Studies

Carolina Guidolin ^1^, Johannes Zauner ^2^, Manuel Spitschan ^2^

^1^  Max Planck Institute for Biological Cybernetics, Tübingen, Germany
^2^  Technical University of Munich, Munich, Germany

**Abstract:Background**: Ocular light exposure significantly influences human circadian and neuroendocrine functions. The emergence of wearable light loggers has enabled studying the effects of light exposure on human physiology under real-world conditions. Participants usually wear light loggers over several days to months and completedaily questionnaires on health outcomes of interest. While this approach allowed inferring day-to-day associations between light exposure patterns and health outcomes, participants often needto remove the light loggers, resulting in non-wear time intervals of varying and unknown durations. Accurate detection of these intervals is essential, particularly when assessing the physiological effects of light and measuring compliance to light therapy regimes in clinical trials. Currently, there are no standard methods for identifying non-wear intervals in light exposure field studies. Here, we deployed a multimodal approach to collect non-wear time information during a longitudinal light exposure campaign and compared the different strategies to inform the best practices for the collection, detection, and handling of non-wear time in ambulatory light exposure studies. **Methods**: Healthy participants (n = 26; mean age 28 ± 5 years, 14F) wore a near-corneal plane light logger (ActLumus, Condor Instruments) measuring melanopic equivalent daylight illuminance (mEDI), mounted on non-prescription glasses, for one week. Participants reported (non-)wear events including taking the light logger off, placing the light logger back on, and taking the light logger off before sleep. These events were logged in three ways, generating independent sources of non-wear information: (1) manually entering the times on an app-based wear log, (2) pressing an event button on the light logger, and (3) placing the light logger in a black bag during non-wear time (leading to <1 lux mEDI light levels). Wear log entries were visually checked twice a day to ensure data quality and used as ground truth for non-wear interval detection. Based on the presence of button press events at either end of a non-wear interval, each interval was automatically classified as “open” or “closed”. **Results**: Based on wear log entries, participants spent 59.2 ± 9.0% (mean ± SD, min. 41.1%, max. 78.0%) of their total participation time wearing the light logger, 6.3 ± 4.3% (mean ± SD, min. 0.4%, max. 15.8%) not wearing the light logger, and 40.1 ± 8.3 (mean ± SD, min. 22.3%, max. 58.9%) with the light logger off during sleep. When considering button presses within a 1 min window from each end, 86.8% of non-wear intervals were classified as closed. Expanding the window size to a more liberal criterion of 8 min increased this number to 91.6%. **Conclusions**: Our analysis shows variability in the duration of the three (non-)wear states across participants. Furthermore, not all wear log-detected non-wear intervals were labeled by a button press event at each end. Thus, relying solely on the presence of button presses to indicate the start or end of a non-wear period would result in misclassification of non-wear time, when the wear log is used as ground truth. Further analyses are ongoing to ascertain whether using clusters of mEDI < 1 lux (i.e., use of the black bag) would identify non-wear intervals accurately. Looking ahead, our detailed dataset will be useful for developing robust, data-driven non-wear algorithms.

**Keywords**: light dosimetry; wearable light loggers; non-wear detection

### 2.16. Thalamocortical Resting State Functional Connectivity in Total Sleep Deprivation and Chronic Sleep Restriction

Aleksandra Domagalik ^1^, Halszka Oginska ^2^

^1^  Centre for Brain Research, Jagiellonian University in Krakow, Kraków, Poland
^2^  Department of Cognitive Neuroscience and Neuroergonomics, Institute of Applied Psychology, Jagiellonian University, Krakow, Poland

**Abstract:Background**: Sleep deprivation, regardless of its cause (sleep disorders, work schedules, lifestyle), results in adverse effects on daytime awake functioning and well-being. The neural background of those effects is not fully understood. It is assumed that thalamus (a key region for communication between cerebral structures) may be responsible for cognitive decrements. Here, we compared the connectivity of thalamus and cortical regions during spontaneous activity in total sleep deprivation and chronic sleep restriction. **Methods**: Twenty-nine young and healthy volunteers (16 females, mean age 23.43 ± 3.65 years) participated in this study comprising three conditions—baseline, one-night total sleep deprivation (TSD), and 5-day (chronic) sleep restriction to 5 h (CSR)—in a counterbalanced order. Participants’ activities were monitored by motion loggers (MotionWatch8, Camntech Ltd.) used on a constant 24/7 basis. Subjective well-being was assessed with use of the Karolinska Sleepiness Scale, CHICa scale, and Positive and Negative Affect Schedule. In each condition, functional magnetic resonance imaging was performed in a 3T scanner (Magnetom Skyra, Siemens). During the scanning resting state (fMRI-rs) data were acquired for 8 min; additionally, the structural image and field map were recorded. Preprocessing was carried out using fMRIprep toolbox (ver. 23.2.0) and later analysis was conducted using AFNI software (ver. 23.1.10). Seed-based correlation was performed on left and right thalamus as a region-of-interest. The ANOVA test was used to verify clusters that showed differences between experimental conditions; the threshold was set to *p* = 0.05. **Results**: Average time-in-bed in baseline conditions was 7 h 56 min (SD 43 min); it was curtailed to 5 h 2 min (SD 17 min) in CSR. The mean of total awake time in TSD was 26 h 3 min ± 141 min. In both deprivation conditions (TSD and CSR), in comparison to baseline, we observed significant increases in sleepiness, CHICa symptoms, and negative affect, as well as a decrease in positive affect (all *p* < 0.001); however, TSD-related subjective symptoms were more severe. FMRI-rs data analysis showed broad connectivity of thalamus to subcortical and cortical areas. The difference in both left and right thalamic connectivity was found in the left and right lateral middle frontal cortex (significant decrease in TSD). Additionally, the connectivity of the left thalamus and primary visual cortex was increased in TSD and CSR conditions. **Conclusions**: As far as subjective well-being is concerned, total sleep deprivation seems to be a more difficult experience than chronic sleep restriction. The decline of functional connectivity between the thalamus and the frontal cortex may be the source of disruption of higher-order functions in a sleep-deprived brain. Those findings also support the hypothesis of a specific vulnerability of the thalamocortical circuits to total sleep deprivation.

**Keywords**: functional magnetic resonance imaging; functional connectivity; thalamus; total sleep deprivation; chronic sleep restriction

**Acknowledgment**: The research was supported by the grant from the Polish National Science Centre No 2018/29/B/HS6/01934.

### 2.17. Exploring Subjective Bias in Lighting Studies: Insights from Systematic Literature Review on EEG Metrics and Study with Metameric Lights

Elifnaz Gecer, Yvonne de Kort, Karin Smolders

Human Technology Interaction group, department of Industrial Engineering and Innovation Sciences, Eindhoven University of Technology, North Brabant, The Netherlands

**Abstract:** (**1**) **Background**: Electroencephalogram (EEG) is a technique to assess the effects of light in an objective manner. Yet while in theory EEG markers may serve as valuable tools for quantifying the non-image-forming (NIF) effects of light, the current findings are inconclusive. Moreover, top-down processing of the sensory stimulus often gets overlooked but may also modulate brain responses beyond bottom-up processing. The lack of proper blinding in lighting studies creates the risk of subjective bias, in the form of predispositions and personal preferences regarding the visible features of light. (**2**) **Methods**: To investigate the potential of EEG as a robust assessment of NIF effects of light during daytime, we first systematically examined light-induced modulations in wake EEG power densities reported in the literature with over 5000 entries from 3 databases, supported by backward and forward search. In tandem with the literature review, we conducted an empirical study involving a 64 channel EEG, metameric light pair (212 lx, 55 lx mEDI, 2097 K vs. 211 lx, 175 lx mEDI, 1773 K), and dim light as the control (9 lx, 2.5 lx mEDI, 2141 K), with broad exploration of EEG markers. Metameric light conditions were calculated based on the Silent Substitution Method and formulated as an optimization problem. We used an in-house engineered Lightbox containing 11 independent LEDs with tunable spectra. (**3**) **Results**: As the findings in the literature regarding EEG frequency metrics are not uniform, we wanted to follow a novel method posing no a priori hypothesis about the location or timing of the effects, nor of the definition of the frequency bands. This ambition came with the challenge of the multiple comparisons we needed to make due to the complex and high spatial, temporal, and spectral resolution of the data, which we overcame by means of applying cluster permutation tests. The systematic literature review revealed an implicit formula of alertness that seemed to be applied in lighting research. This formula pointed to an increase in alertness if there was a reduction in the lower frequency power (such as alpha, delta, theta) and if there was an increase in higher frequency power (such as beta or gamma). In the empirical study containing metamers with almost identical looks, we were able to replicate findings between dim control condition and (brighter) metameric conditions, but not between the metameric conditions themselves (low melanopsin and high melanopsin), except for alpha power (Monte Carlo *p* = 0.03). (**4**) **Conclusions**: It remains essential to highlight the inconsistencies present in the existing literature as well as the need for a replication of the findings. Existing daytime EEG signal metrics often fall short, highlighting the value of exploring underutilized EEG metrics to quantify the NIF effects of light during daytime. This presentation will delve into the possible mechanism behind subjective biases and propose methodological improvements to mitigate them in future research and practical applications.

**Keywords**: NIF effects; systematic literature review; EEG; metameric light; melanopsin; alertness; permutation testing

**Funding:** The LightCap project has received funding from the European Union’s Horizon 2020 research and innovation programme under the Marie Skłodowska-Curie grant agreement No. 860613.

### 2.18. The Potential of Light Dosimetry in Field Studies: Insights from Data Collected in School, Office, and Healthcare Environments

Marilyne Andersen, Steffen Hartmeyer

EPFL, Écublens, Switzerland

**Abstract:** (**1**) **Background***:* By now it is well known that daily light exposure influences circadian rhythms, sleep, alertness, mood, and many other physiological and psychological functions. While existing and emerging recommendations regarding “good” lighting practices to support human health and well-being differ in their content, most insist on the importance of the light’s spectral composition, the need for bright days and dim nights, and the benefits of sufficient daylight exposure. Our daily exposure to light can be monitored with the help of wearable light sensors (light dosimetry), offering unique insights into how personal light exposure is affected by individual lifestyles and environmental characteristics, and how light exposure, in turn, impacts health-related outcomes. (**2**) **Methods***:* To investigate the potential of light dosimetry for future field studies, three studies were conducted to collect spectrally resolved light exposure data over multiple days in different real-life environments as part of a light-intervention study in an education context (between-subjects design in school classrooms in Reykjavik with 17 teenagers) and a work context (within-subjects design in an open-plan office in London with 20 adult employees), and as part of an observational study in a healthcare context (within-subjects design with 55 shift workers in a hospital in Geneva). Data were collected with the *Spectrace* dosimeter prototype, measuring spectral irradiance across 18 channels (14 in the visible range and 4 in the near-infrared range). The collected data were processed to characterize and compare the patterns of exposure to different quantities and spectral distributions of light experienced by the participants in their daily lives. This processing step included a novel approach to cluster and classify the spectral data, using various reference spectra from publicly available datasets to differentiate between known spectral types. (**3**) **Results***:* The primary aim of the three studies was to gain a better understanding of the “spectral diets” of the participants, both inside and outside of the “controlled” time windows (i.e., while at school or work). The results showed the potential of spectrally resolved light dosimetry to differentiate the dominant spectral types experienced over the course of the day, allowing to validate experimental conditions, assessing the proportion of time spent in daylight, and examining the impact of individual behavior. Moreover, by relating light exposure metrics such as the frequency of light levels, the time above threshold, and cumulative light exposure to collected responses (e.g., subjective sleep parameters), potential relationships between light exposure and physiological or behavioral outcomes could be examined. (**4**) **Conclusions***:* The aim of these studies was to demonstrate that it is feasible to measure and identify the types of light spectra people are individually exposed to over time and ultimately to relate these “spectral diets” to potential health outcomes. Notably, this approach revealed how much can be learned about the impact of individual behavior “just” from measured light exposure and provided new insights on the impact of lighting conditions in a given environment (e.g., the workplace) relative to the overall daily light dose.

### 2.19. Protocol for Evaluating the Usability, Acceptability, and User Satisfaction of an mHealth Behavior Change Intervention to Optimize Light Exposure Among Older Adults

Resshaya Roobini Murukesu ^1,2^, Resshaya Roobini Murukesu ^1,2^, Denz Del Villar ^1,2^, Manuel Spitschan ^2^

^1^  TUMCREATE
^2^  TUM School of Medicine & Health, Department of Health and Sport Sciences, Tech-nical University of Munich, Munich, Germany

**Abstract:Background:** Light is a key environmental stimulus for regulating circadian rhythms, sleep, mood, cognition, physical health, and eye health. Despite the well-documented importance of light exposure for aging health, translating this knowledge into targeted interventions to support healthspan and longevity still needs to be explored. This observation is particularly relevant within preventative health and medicine. With the rapid increase in the global aging demographic and migration towards digital health technologies, there is a need to support lifestyle-based aging-in-place strategies, particularly via targeted mobile health (mHealth) interventions addressing the importance of optimized light exposure for aging health and well-being. **Objective**: This pilot study aimed to assess the usability, acceptability, and user satisfaction of the LightSPAN mHealth smartphone application (app) that integrates behavior change strategy and output from wearables to optimize healthy light exposure among older adults in Singapore. **Methods**: This pilot study used a randomized, controlled, 5-week, 2-period, crossover design. Generally healthy older adult smartphone users aged 60 years and above (target n = 20) were recruited from established Active Aging Centers in Singapore. Participants were given two wearables: a pendant-attached light sensor to capture daily light exposure (ActLumus, Condor Instruments, São Paolo, Brazil) and a consumer-grade wrist-worn fitness tracker for sleep and health monitoring. Participants were allocated to two groups: for the first two weeks, one group started with the digital intervention (DI), receiving the LightSPAN mHealth app, whereas the other group started with the digital placebo (DP) app which mimics the DI app while omitting the active component of behavior change and wearables’ feedback. After a one-week washout, the groups were switched from DI to DP and vice versa for the next two weeks. Assessments occurred twice, at the end of weeks 2 and 5. Usability and user satisfaction of the LightSPAN mHealth app were assessed with the mHealth App Usability Questionnaire (MAUQ) for standalone mHealth Apps. Acceptability was evaluated using the Technology Acceptance Model (TAM) adapted to the context of the LightSPAN mHealth app. Acceptability was further explored qualitatively via post-pilot discussion and contextual inquiry with the participants to obtain perspectives on user experience, preferences, challenges faced, and participants’ perceptions regarding the DI and DP for iterative improvement. **Conclusions**: This study determined the usability, acceptability, and user satisfaction of the LightSPAN mHealth app among older adults. These findings provided valuable insights, enabling the fine-tuning of the LightSPAN mHealth app and digital placebo prototypes to enhance effectiveness and usability in optimizing light exposure via mHealth. Moreover, this study addressed the technical and behavioral aspects of app usage. It also provided a comprehensive understanding of the interaction between technology and older adults, paving the way for delivering a tailored, user-centric, and personalized digital health intervention.

**Funding:** National Research Foundation Singapore (NRF2022-THE004-0002).

### 2.20. Disturbance of Hormonal Circadian Rhythms by Light Pollution

Michal Zeman

Department of Animal Physiology and Ethology, Faculty of Natural Sciences, Comenius University, Bratislava, Slovakia

**Abstract:** The neuroendocrine system coordinates physiological and behavioral processes with acute environmental conditions and represents a key component of allostasis. Synchronization of the neuroendocrine system with the rhythmic environmental changes is enabled by the circadian system. Most hormones exhibit distinct daily rhythms, which are either directly controlled by the central circadian oscillator localized in the suprachiasmatic nucleus (SCN) of the hypothalamus or indirectly, reflecting daily behavioral and metabolic rhythms. The SCN is entrained by the light (L)-dark (D) cycle, which used to be the extremely reliable environmental variable for millions of years and has been disturbed only recently by human-produced artificial light at night (ALAN). ALAN is a new environmental risk factor associated with negative effects on biodiversity and health, which are attributed to disruption of the circadian timing system. Deregulation of circadian rhythms is considered the common risk factor of the high incidence of diseases of civilization, such as metabolic, cardiovascular, and neurological diseases, but underlying mechanisms remain poorly understood. In our research, we focused on the neuroendocrine system as the key control system which can mediate effects of circadian disruption on physiology and behavior. Our data show that exposure to dim ALAN (2 lx) causes in male rats a disruption of circadian control of the neuroendocrine system as proved by strong attenuation of the molecular clockwork in the SCN, as indicated by the damped and lost daily rhythmicity in the clock genes Per1 and Per2, respectively. Moreover, rhythmic expression of Nr1d1 and arginine vasopressin (Avp) damped, and we observed disturbed rhythmic clock gene expression in the paraventricular and dorsomedial hypothalamic nuclei that convey the circadian signals from the SCN to endocrine and behavioral rhythms. Disruption of these output pathways in ALAN-exposed rats was manifested by lost daily variations in plasma melatonin, testosterone and AVP concentrations, and by the damped and phase-advanced plasma corticosterone rhythm. These changes are associated with a disturbed daily pattern of metabolites and behavioral rhythms in activity and food and water intake suggesting a disturbed circadian control of metabolism, immunity, and the control of the cardiovascular system. In addition to the neural connections, the SCN can communicate circadian signals via diffusible factors, mainly AVP, which connects via a special portal system the SCN with the organum vasculosum of the lamina terminalis (OVLT) involved in osmotic control. Studies in mice have shown that AVP via the OVLT ensures the circadian control of anticipatory thirst before sleep. Interestingly, the pronounced increase in water intake during the last part of the active period, which protects against dehydration in the absence of drinking during sleep, was compromised by exposure to dim ALAN. Moreover, simultaneous exposure of rats to ALAN and high fructose intake additively inhibits melatonin biosynthesis, opening the possibility that ALAN in cooperation with other risk factors can negatively affect the neuroendocrine control of physiology and behavior with negative consequences on health.

**Funding:** Supported by APVV-21-0223.

### 2.21. Circadian Dynamics in an In Vitro Model of Cutaneous Wound Repair

Gemma Farrington

Liverpool John Moores University, Liverpool, UK

**Abstract:** Circadian rhythms govern daily oscillations in behavior, metabolism, and physiology. These oscillations are driven by endogenous cellular clocks and underpinned by the rhythmic expression of a set of core circadian clock genes, proteins, and metabolites. Disruption of the circadian system is linked to chronic disorders such as cardiovascular disease, diabetes, and impaired wound healing. In the skin, dysregulation of wound repair arises from the altered behavior of epidermal keratinocytes and dermal fibroblasts as well as immune cells, all of which must function together to orchestrate wound closure in a coordinated manner. Chronic wounds are associated with increased inflammation and reduced proliferation of fibroblasts and represent an enormous social and economic burden to the healthcare system. Wound repair processes are known to be controlled by microRNAs (miRNAs). These short non-coding RNAs mediate post-transcriptional regulation of gene expression and influence the different stages of wound healing, including inflammation, proliferation, and remodeling. Moreover, miRNAs have been implicated in the regulation of circadian rhythms through their effects on core clock genes. However, whilst it is clear that both miRNA expression and circadian activity are important for wound repair in skin, we currently know little about circadian function in chronic wounds, or interactions between circadian processes and miRNA expression in this context. My research will determine whether circadian function and miRNA expression are altered in immortalized human dermal fibroblasts derived from chronic wounds, investigate interactions between these important physiological control mechanisms, and evaluate the impact of circadian variables on the wound healing responses driven by miRNAs. This research will thus provide mechanistic insights that integrate circadian biology with the exciting potential of RNA-directed therapeutic interventions to promote the healing of chronic wounds.

### 2.22. The Effects of Total Sleep Deprivation on the Circadian Rhythms and Psychophysiological Factors of Military Cadets: A Comparison Between Wakefulness in Light and Darkness

Katka Skálová ^1^, Jan Maleček ^2^, David Kolář ^3^, Jana Kopřivová^3^, Zdeňka Bendová ^1^

^1^  Faculty of Science, Charles University, Staré Město, Czech Republic
^2^  Faculty of Physical Education and Sport, Charles University, Staré Město, Czech Republic
^3^  National Institute of Mental Health, Prague, Czech Republic

**Abstract:** Total sleep deprivation (SD) significantly impairs cognitive function, memory, decision-making, emotional stability, socio-emotional functioning, and influences hunger and subjective taste preferences. Sleep deprivation, but also nighttime light, have negative effects on human health and performance. The presence or absence of light at night can divide SD into two scenarios: wakefulness under the influence of light, as experienced during night shifts, and wakefulness in the dark, as when lying in bed unable to fall asleep due to some stressor. The aim of our study was to compare the immediate and transient effects of total, 39 h SD under different lighting conditions: (1) we simulated full indoor lighting similar to shift work, and (2) darkness reminiscent of stress-induced insomnia. We conducted our study with 18 volunteer military students. Of these, 18 underwent SD with light (SD/L) and 12 underwent SD in the dark (SD/D). We measured melatonin levels during SD nights, locomotor activity, peripheral temperature rhythm profiles, cognitive performance, mood, hunger, and food preference before and after SD and after recovery sleep. Our data show that peripheral temperature rhythm and post-SD activity returned to baseline faster after SD in darkness. Subjective sleepiness increased after SD, with light at night exacerbating morning sleepiness. Positive affect decreased after SD and returned to baseline after recovery sleep. Cognitive performance was better after SD in the light, but satiety and post-breakfast blood glucose were higher after recovery sleep after SD in the dark, and sweet taste preference was significantly higher after SD in the dark. Our results suggest that light exposure during SD may reduce fatigue the next day. However, the effects on the circadian system are more pronounced with light during SD, leading to persistent changes in diurnal rhythms of temperature and activity. Our research emphasizes the contrast between the immediate benefits and potential lasting detriments of nighttime light exposure to health, particularly in relation to effects on the circadian system, a consideration relevant to worker management practices.

### 2.23. The Circadian Modulation of Melanin Biosynthesis by Targeting the ROR Regulatory Motif in the Retinal Pigment Epithelium

Nemanja Milicevic ^1^, Amandine Bery ^2^, Bobin George Abraham ^1^, Marie-Paule Felder-Schmittbuhl ^2^, Arthur A. Bergen ^3^, Teemu Ihalainen ^1^, Soile Nymark ^1^

^1^  Tampere University, Tampere, Finland
^2^  University of Strasbourg, Strasbourg, France
^3^  Amsterdam UMC, Amsterdam, The Netherlands

**Abstract:Background**: Albinism is a pigment disorder characterized by deficiency of melanin pigment in the eyes, skin, and/or hair. Melanin is synthetized in complex biochemical pathways which are not fully characterized. We hypothesized that these pathways are regulated by the circadian clock. The circadian clock is a molecular pacemaker comprising transcriptional and translation feedback loops involving transcription factors such as BMAL1, CLOCK, PER1-2, CRY1-2. Our goal was to investigate the link between the circadian clock and melanogenesis. **Methods**: We cultured embryonic stem cell-derived retinal pigment epithelium (hESC-RPE) for 12 weeks. The cells were synchronized by a serum-shock and harvested at 3 h intervals for up to 66 h. RT-PCR analysis was used to quantify the expression of clock and melanogenesis genes. We performed a motif-enrichment search in melanogenesis genes by using online tools (*UCSC* database, Galaxy, and TF Motif View). Pigmentation in hESC-RPE was studied using pharmacology, bright-field microscopy and analyzed by an AI-based trainable WEKA segmentation tool. Bioinformatics was used to analyze published RNA-sequencing data of laser capture microdissected mouse RPE obtained at 4 time points over 24 h. **Results**: We found that synchronized hESC-RPE show rhythmic mRNA expression of clock genes *BMAL1*, *CLOCK*, *CRY2*, *PER1*, *PER2,* and *REV-ERBα* and melanogenesis-related genes *TYR*, *CDH3*, *DCT*, and *PMEL.* We selected mouse and human conserved promoter regions by using the *UCSC* database with 6 known time-affected melanogenesis genes (*Tyr*, *Tyrp1*, *Pmel*, *Dct*, *Cdh3*, and *Rab1a*). By using the motif-enrichment tool, TF Motif View, we found over-representation of binding sites for negative regulators of transcription and the ROR binding site, a known regulator of clock genes. Bright-field microscopy revealed decreased pigmentation in hESC-RPE treated with the ROR agonist Nobiletin compared with the controls. Conversely, significantly increased pigmentation was observed in hESC-RPE cells supplemented with the indirect ROR antagonist SR9009 compared with the controls. Finally, bioinformatics analysis revealed that melanogenesis pathways are enriched in genes upregulated at night compared with late-afternoon time points in mouse RPE. **Conclusions**: Overall, these results suggest that melanogenesis is regulated by the circadian clock. Modulation of the clock could be a potential target for treating albinism.

**Keywords**: clock genes; circadian rhythm; retinal pigment epithelium; melanogenesis; melanin; stem cell

**Funding:** Academy of Finland (decision: 340127), Finnish Cultural Foundation (Finland), the Federation of European Biochemical Societies (FEBS) Short-Term Fellowship (EU), the Centre National pour la Recherche Scientifique, and the University of Strasbourg (France).

### 2.24. Determinants of Sleep Under Real-World Conditions: Preliminary Results from the Longitudinal, 12-Month Ecology of Human Sleep (EcoSleep) Cohort Study

Anna M Biller ^1^, Nayab Fatima ^1^, Chrysanth Hamberger ^1^, Laura Hainke ^2^, Verena Plankl ^1^, Amna Nadeem ^1^, Achim Kramer ^3^, Martin Hecht ^4^, Manuel Spitschan ^1^

^1^  Technical University of Munich, Chronobiology & Health, TUM School of Medicine and Health, Munich, Germany,
^2^  Technical University of Munich, TUM School of Medicine and Health, Department of Psychiatry and Psychotherapy, Munich, Germany
^3^  Charité-Universitätsmedizin Berlin, Laboratory of Chronobiology, Berlin, Germany
^4^  Helmut Schmidt University, Department of Psychology, Hamburg, Germany

**Abstract:Introduction**: To enable precision prevention in sleep and circadian health, it is key to understand individual variability in sleep and light exposure and derive individual-level determinants. In the Ecology of Human Sleep (EcoSleep) Project (protocol preprint: https://doi.org/10.1101/2024.02.09.24302573), we sampled daily life factors, sleep, circadian rhythms, and light exposure over 12 months to understand which factors influence the subsequent sleep episode and characterize inter- and intra-individual variability of sleep and light exposure long-term. **Methods**: The target sample size was N = 12 (50% female) healthy students. Data collection began in mid-March 2024 with n = 9 participants enrolled (67% female). Continuous measurements included personal melanopic light exposure using a pendant-worn light logger, wrist-worn actimetry and light logging, bedroom environmental variables (ambient light, temperature, humidity, and air pressure), and continuous glucose measurements (CGM). During monthly three-day measurement sessions, ambulatory electroencephalograms (EEG) were recorded at home, smartphone-enabled ecological momentary assessments took place four times/day, distal–proximal skin temperature gradient (DPG) was measured continuously, and saliva and hair samples were taken for circadian phase and amplitude. Monthly questionnaires included a chronotype assessment and Light Exposure Behavior Assessment (LEBA). **Results**: Nine participants aged 21–33 (mean 26 ± 3.5 years; 67% female) recorded up to 13 weeks of continuous data. The EEG data suggest no average first-night effect but high intra-individual variability between first and second EEG nights, corroborated by individual subjective sleep diary data. In the DPG recordings, a warmer proximal compared with distal skin temperature during sleep was observed, demonstrating the feasibility of DPG in field studies. CGM provided mostly high-quality data ranging in physiologically healthy levels (i.e., 70–100 mg/dL, 140 mg/dL after meals). LEBA data show that individual scores on the five factors describing light behaviors ranged almost on the entire scale (i.e., high individual variance) except for Factor 2 (“Spending time outdoors”), in which participants averaged 66% indicating longer or more frequent self-reported light exposure outside than average. Individual items suggested low use of blue-blocking glasses indoors and outdoors or before falling asleep (77–85% indicated “never”) and mixed use of LED or desk lamps. In contrast, frequent use of smartphones 1 h prior to sleep (71% “always or often” and 29% “sometimes”) and immediately after wake but not at night when waking was reported (71% “rarely or never”, 29% “sometimes”). Participants also reported frequent use of phone screen dimming or blue filter use on their laptops. Only two participants reported “always” walking outside within 2 h hours after getting up, which did not seem to completely explain their chronotype (one earlier, one later chronotype). Most participants reported spending as much time outside as possible (1–3 h or less). **Conclusions**: Our preliminary analyses indicate the feasibility of the EcoSleep protocol. Subjective light data suggest that the cohort seems to prioritize healthy light environments and spend as much time outside as possible. Further analyses include data relating subjective behavior to objective light exposure from both light logging sources to (i) validate subjective instruments, (ii) to understand variability in different wearing locations, and (iii) to relate day-to-day subjective light exposure to sleep-related outcomes.

### 2.25. Sleep for Attention by Attention to Sleep: Treatment of Sleep Disorders in Adults with ADHD

Mirte van der Ham ^1^, Denise Bijlenga ^1^, Danielle Starreveld ^1^, Mylène Böhmer ^1^, Ravian Wettstein ^2^, Glenn Dumont ^2^, Aartjan Beekman ^3^, Sandra Kooij ^1^

^1^  PsyQ Expertise Center Adult ADHD, Amsterdam, The Netherlands
^2^  ADHDcentraal, Utrecht, The Netherlands
^3^  Amsterdam UMC, Vrije Universiteit, Psychiatry, Amsterdam Public Health research institute, Amsterdam, The Netherlands

**Abstract:Background**: Sleep problems and disorders are highly prevalent in adults with attention-deficit/hyperactivity disorder (ADHD). The most commonly diagnosed sleep disorder in these patients is the delayed sleep phase syndrome (DSPS), characterized by an, on average, 1.5 h delay in dim light melatonin onset (DLMO) in 78% of adults with ADHD. Other sleep disorders, such as insomnia and restless legs syndrome/periodic limb movement disorder (RLS/PLMD), are also much more prevalent compared with the general population. Sleep problems and ADHD symptoms are intertwined, where they can cause, amplify, and maintain each other. We aimed to study the effects of both ADHD treatment and sleep treatment (separately and combined) on ADHD and sleep parameters in adults with ADHD and sleep problems. **Methods**: Seventy adult patients diagnosed with ADHD and a positive screening on the Holland Sleep Disorders Questionnaire (HSDQ) for at least one sleep disorder were included in this open-label randomized controlled trial. Participants were randomized for a 12-week period of (1) sleep treatment, (2) ADHD treatment, or (3) a combined sleep and ADHD treatment. Sleep treatment consisted of chronotherapy for a delayed circadian rhythm, cognitive behavioral therapy for insomnia, and if indicated, additional treatment for other sleep disorders. ADHD treatment consisted of standard pharmacological treatment and psychological coaching. Assessments were carried out at baseline (T0) and after 6 and 12 weeks of treatment (T1 and T2, respectively) and consisted of a nocturnal polysomnography, objective and subjective ADHD symptoms (the QbTest and ADHD Rating Scale, respectively), subjective depressive symptoms, and sleep symptoms. **Results**: Analysis of the impact of the interventions on ADHD symptoms, sleep, and other outcomes are currently ongoing. Preliminary paired samples t-tests on ADHD outcomes showed a significant decrease in subjective ADHD symptoms between T0 and T2 in the sleep treatment group (t = 3.40, *p* < 0.01) and the combined treatment group (t = 7.16, *p* < 0.001). Objective activity, impulsivity, and inattention parameters decreased significantly in the combined treatment group (t = 3.38, *p* < 0.01; t = 2.90, *p* = 0.01; t = 4.086, *p* < 0.001, respectively). Preliminary multilevel analyses showed no significant differences in treatment effects between standalone ADHD treatment and combined treatment on subjective and objective ADHD symptoms. Sleep treatment alone also reduced objective ADHD symptoms, but to a lesser extent than standalone ADHD treatment and combined treatment. **Conclusions**: Both standalone sleep treatment and a combined ADHD and sleep treatment are able to reduce ADHD symptoms in adult patients with ADHD.

### 2.26. Day-to-Day Dynamics of Sleep Quality and Affect in Individuals with a Major Depressive Episode Undergoing Bright Light Therapy

Jasmijn de Joode, Emma Visser, Yvonne de Kort

Human Technology Interaction group, department of Industrial Engineering and Innovation Sciences, Eindhoven University of Technology, North Brabant, The Netherlands

**Abstract:Background**: Bright light therapy (BLT) is an effective treatment for depression, involving exposure to bright light at 10,000 lux for at least 30 min per day for multiple weeks. Despite its efficacy, the mechanisms underlying BLT’s antidepressant effects are not fully understood. While studies suggest that BLT influences mood, sleep, and circadian rhythms, the complex interplay between these factors complicates determining its precise effects. This study aimed to unravel part of these dynamics by investigating the day-to-day relationships between sleep quality and affect in individuals with a major depressive episode undergoing BLT. **Methods**: This research is part of the BioClock Study, a randomized controlled trial investigating the optimization of BLT for depressive disorders (ClinicalTrials.gov ID NCT05958940). Participants received one to three weeks of BLT, during which variations in positive and negative affect were assessed eight times per day using an ecological momentary assessment. Sleep quality was evaluated every morning using the Consensus Sleep Diary. To examine the bidirectional and autoregressive relationships between sleep quality and affect, we employed multilevel structural equation modeling. All variables were centered within persons reflecting the deviation from the personal mean. Subsequently, all variables were lagged by one day to assess cross-lagged and autoregressive relationships. **Results and Conclusions**: Preliminary results from the first 12 participants will be presented at the conference. These findings will provide initial insights into the intricate mechanisms of BLT, potentially leading to greater clinical adoption of the therapy in the future.

**Keywords**: bright light therapy; depression; ecological momentary assessment; multilevel structural equation modelling; affect; sleep quality

**Funding:** This work is part of the BioClock project (with project number 1292.19.077) of the research program Dutch Research Agenda: Onderzoek op Routes door Consortia (NWA-ORC) which is (partly) financed by the Dutch Research Council (NWO).

### 2.27. How Prenatal LPS and Early-Life Constant Light Exposure Alter Circadian Gene Expression Profiles in Different Rat Tissues

Veronika Spišská, Aneta Kubištová, Zdeňka Bendová

Department of Physiology, Faculty of Science, Charles University, Prague, Czech Republic

**Abstract:** Prenatal exposure to lipopolysaccharide (LPS) leads to neurobiological and behavioral changes. Likewise, continuous exposure to constant light (LL) during the critical developmental period of the circadian system alters gene expression in many tissues in adulthood. In our study, we primarily investigated the individual effects of both interventions and, more importantly, their combined effect. We aimed to determine if there might be a synergistic effect of those interventions on circadian rhythms of various gene expressions. We focused mainly on clock genes, immune-related genes, and specific genes in the hippocampus, pineal gland, spleen, and adrenal gland of rats at postnatal day 30. Our results show a significant influence of prenatal LPS or postnatal LL on the expression profile of all assessed genes. However, combined prenatal LPS and postnatal LL only enhanced the negative effect in the minority of the comparisons. In most cases, combined intervention appeared to attenuate the changes induced by the individual intervention, restoring the measured parameters closer to those of the control group. Our data suggest that a mild immunological challenge during prenatal development may trigger an adaptive response of the circadian clock later in life.

**Keywords**: circadian clock; constant light; lipopolysaccharide

**Funding:** This work was funded by the Charles University Grant Agency (grant no. 370121), the Czech Science Foundation (grant no. 23-07184S), and the European Regional Development Fund-Projects “PharmaBrain” No.CZ.CZ.02.1.01/0.0/0.0/16_025/0007444.

### 2.28. Sleep and Circadian Activity Rhythms During Immunotherapy Treatment in Lung Cancer Patients Are Prospectively Associated with Fatigue, Disease Progression, and Survival

Louise Strøm ^1^, Robert Zachariae ^2^, Lisa Maria Wu ^3^, Peter Meldgaard ^4^, Sonia Ancoli ^5^, Mats Lekander ^6^, Daniel Mroczek ^7^, Ali Amidi ^1^

^1^  Sleep & Circadian Psychology Research Group, Department of Psychology & Behavioural Sciences, Aarhus University, Aarhus, Denmark
^2^  Unit for Psychooncology & Health Psychology, Department of Oncology, Aarhus University Hospital, Aarhus, Denmark
^3^  Department of Psychology, Reykjavik University, Reykjavík, Iceland
^4^  Denmark-Department of Oncology, Aarhus University Hospital, Aarhus, Denmark
^5^  Department of Psychiatry, University of California San Diego School of Medicine, Israel, La Jolla, CA, USA
^6^  Division of Psychology and Osher Center for Integrative Health, Department of Clinical Neuroscience, Karolinska Institutet, Stockholm County, Sweden
^7^  Department of Medical Social Sciences, Northwestern University Feinberg School of Medicine, Chicago, IL, USA

**Abstract:Background**: Lung cancer, including non-small cell lung cancer (NSCLC), is among the most common cancers worldwide and currently the leading cause of cancer-related deaths. In recent years, there has been a shift towards targeted therapies and immunotherapies employing checkpoint inhibitors (ICIs), with notable improvements in survival rates. These therapies act through their activation of the immune system but may potentially result in disrupted sleep and circadian rhythms with negative implications for behavioral and psychological symptoms such as fatigue, depression, and stress. Furthermore, disrupted sleep and circadian rhythms may possibly underlie premature treatment discontinuation and poorer prognosis. Improved understanding of potential modifiable factors such as sleep and circadian rhythms could assist clinicians in timely interventions, preventing treatment discontinuation, improving quality of life, and possibly even prognosis. The aims of the present study were, thus, to prospectively examine the trajectories of sleep and circadian activity rhythms in NSCLC patients undergoing treatment with ICIs and to investigate associations with symptoms of fatigue, depression, and stress, as well as prognostic outcomes. **Methods**: Newly diagnosed NSCLC patients referred to the Department of Oncology at Aarhus University Hospital between September 2019 and December 2021 were invited to participate in this preregistered, prospective study. Circadian activity rhythms were assessed continuously with actigraphy (ActTrust) for a period of five months. Weekly assessments of insomnia severity were undertaken with the Insomnia Severity Index during the same period. Finally, validated measures of psychological symptoms (fatigue, depression, stress) together with sleep diaries were obtained every three weeks corresponding to every treatment cycle. Actigraphy data were analyzed in one-week periods and circadian robustness for each period was assessed with the Circadian Function Index (CFI). Change over time in sleep and circadian outcomes and longitudinal associations with psychological symptoms were analyzed using mixed linear models. Follow-up data on prognostic outcomes were collected from medical records and included time to treatment discontinuation, progression, and cancer-specific death. Time-to-event analyses were conducted with Kaplan–Meier survival plots and Cox regressions to estimate hazard ratios of the prognostic outcomes. **Results**: Of 65 eligible patients, 49 (75%) were enrolled, and 31 (63%) completed five months of data collection. At baseline, 49% experienced clinical insomnia (ISI ≥ 10) and 68% slept ≤ 7 h a night. Gradual improvements were observed in ISI and stress across the treatment period (*p*s < 0.006). Over time, more robust circadian activity rhythms (CFI) were statistically significantly associated with reductions in fatigue (*p* = 0.013) but not depression or stress. A significant association, over time, was also found between higher levels of insomnia severity and higher levels of fatigue (*p* = 0.017). Cox regression analyses indicated that patients with trajectories of circadian robustness below the median were three times more likely to experience disease progression (HR = 3.75, *p* = 0.005) and death (HR = 3.07, *p* = 0.028). **Conclusions**: Sleep and circadian activity rhythms are important modifiable outcomes that may underlie detrimental behavioral and psychological outcomes during lung cancer treatment. Less robust circadian rhythms may even be predictive of disease progression and survival.

### 2.29. Classification of Natural Indoor and Outdoor Scenes from Radiometric, Photometric, and Colorimetric Features

Niloufar Tabandeh ^1^, Dominique Dumortier ^2^, Claude Gronfier ^3^, Sophie Jost ^2^, Lenka Maierova ^4^, Aiman Raza ^2^, Jennifer A. Veitch ^5^, Manuel Spitschan ^6^

^1^  Max Planck Institute for Biological Cybernetics, Tübingen, Germany
^2^  École nationale des travaux publics de l’État (ENTPE), Vaulx-en-Velin, France
^3^  Institut national de la santé et de la recherche médicale (INSERM), Paris, France
^4^  Czech Technical University in Prague, Prague, Czech Republic
^5^  National Research Council Canada, Ottawa, Ontario, Canada
^6^  Technical University of Munich, München, Germany

**Abstract:Background**: In natural, real-world scenes, environmental light can vary significantly in intensity, spectrum, color, and spatial and temporal characteristics due to the presence of different light sources (daylight, electric lighting, self-luminous displays, and mixtures thereof) and light–surface interactions. The “spectral diet” an observer is exposed to reflects this complexity, compounded by body, head, and eye movements. Understanding some of this complexity requires a systematic and detailed understanding of the variation in light in the real world. From the first principles, we know that light outdoors is of higher intensity than light indoors. A detailed survey of the spectral, spatial, and temporal features of light in the real world, which is highly relevant for architectural design, occupational health, and environmental medicine, has thus far not been undertaken. **Methods**: In the SCENES Dataset (https://www.scenes-dataset.org/, accessed on 30 May 2024), we comprehensively characterized the spectral, spatial, and temporal variations in natural scenes using a novel multimodal data collection setup comprising an α-opic imaging radiometer, a high-resolution spectroradiometer, illuminance and colorimetric measurement devices, a depth camera, and an uncalibrated wide-field RGB video camera. All instruments were integrated into a portable box for easy deployment with a power supply through external batteries. Data were collected across various times of day and seasons. Each scene was described using a novel metadata descriptor (n = 43 items) encompassing detailed information, including geographical information, weather conditions, and scene categories (9 indoor subcategories, 11 outdoor subcategories). To understand the basic aspects of the datasets, we used descriptive statistics (mean, SD, min., max.), and applied the random forest algorithm to develop a scheme for indoor vs. outdoor specifications on spectrum-derived data. **Results**: We measured natural scenes both indoors (n = 313) and outdoors (n = 366) in five locations (Tübingen, Germany: n = 53 indoors, n = 254 outdoors; Munich, Germany: n = 61 indoors, n = 11 outdoors; Prague, Czech Republic: n = 19 indoors, n = 16 outdoors; Lyon, France: n = 67 indoors, n = 26 outdoors; and Ottawa, Canada: n = 113 indoors, n = 59 outdoors). As expected, there were key differences between indoor vs. outdoor scenes in photopic illuminance (mean ± SD 1826.51 ± 7402.82 lx [min. 13.8 lx, max. 63721.1 lx] indoors vs. 13401.91 ± 7402.82 lx [0.28 lx, 110872.8 lx] outdoors) and melanopic equivalent daylight illuminance (mean ± SD 1586 ± 6416.93 lx [min. 11.9 lx, max. 55178.3 lx] indoors vs. 12240.01 ± 18741.81 lx [0.19 lx, 97807.52 lx] outdoors). The results from the random forest algorithm indicate excellent model performance (accuracy: 0.963, precision: 0.982, recall: 0.931, F1: 0.956). Regarding feature importance in the classification, CRI Ra (color rendering index) was ranked highest. **Conclusions**: In this study, we collected indoor and outdoor natural scene data across various geographical contexts. A preliminary analysis evaluated the ability of several spectrum-derived features to allow for the classification of indoor vs. outdoor scenes. Further analyses will investigate the possibility of classifying scene subcategories and probe the spatiotemporal characteristics of natural scenes in further detail. The SCENES Dataset will be made available as an open-access dataset to serve as a benchmark of the properties of environmental light.

**Funding:** Bayerische Forschungsallianz (Bavarian Research Alliance); BayFrance (Bayerisch-Französisches Hochschulzentrum/Centre de Coopération Universitaire Franco-Bavarois) FK-13-2023; and BAYHOST (Bayerisch-Tschechische Hochschulagentur/Česko-bavorská vysokoškolská agentura) BTHA-AP-2023-27.

### 2.30. A Field Study in Denmark Investigating the Biological Impact of Artificial Outdoor Lighting at Night on the Circadian Rhythm of Humans and Animals

Xinxi Zeng ^1^, Thierry Soreze ^1^, Joachim Stormly Hansen ^2^, Paul Michael Petersen ^1^

^1^  Technical University of Denmark, Kongens Lyngby, Denmark
^2^  Ocutune, Roskilde, Denmark

**Abstract:Background**: Light is essential for visual perception of our surroundings, and it has a profound impact on the circadian rhythm in both humans and animals. Until recently, image-forming measuring units were mostly used to characterize both systems. With the publication of CIE S026:2018, melanopic equivalent daylight illuminance (MEDI) and alpha-opic-EDIs were introduced, describing the response of both image-forming and the non-image forming photoreceptors (e.g., ipRGC), now referred to within CIE as Integrative Lighting. These have been widely accepted as key parameters for evaluating the impact of light on human circadian rhythms. With the increasing amount of artificial light at night (ALAN), both humans and other living organisms are experiencing the negative effects of light pollution, with biological and circadian rhythms disrupted. To reduce light pollution and minimize wildlife disruption, the Illuminating Engineering Society (IES) and International Dark-Sky Association (IDA) have adopted “Five Principles for Responsible Outdoor Lighting”, serving as a guideline for outdoor lighting. **Methods**: To assess the photometric and colorimetric characteristics of the outdoor luminaires, together with its potential impact on the biodiversity in the Zealand area of Denmark, measurements (point measurements and datalogging) were carried out at night in 6 locations across 5 municipalities, using a spectroradiometer, mounted on a tripod, with adjustable height. The Luox platform was used to calculate the alpha-opic-EDIs from measured spectra, and the AlphaOpics platform by Lucas et al. 2023 was used to calculate the responses from various species such as rats and horse to measured spectra. **Results**: Our initial finding suggests that the current ALAN in Zealand, Denmark, is potentially disruptive to the circadian rhythm of nearby living animals as the illuminance and MEDI levels from the outdoor luminaires were quite high, and the luminaires appeared to be glary. Our measurements in *Ørestad Syd* showed that blackbirds probably avoid areas with artificial outdoor lighting, as the MEDI measured levels are more than 40 times higher than the threshold for 50% melatonin suppression. Furthermore, the wide light distribution of the luminaires can cause light trespass, illuminating the areas where light is not needed. **Conclusions**: We conclude that to reduce light pollution caused by ALAN and negative impacts on biodiversity, the outdoor luminaires in Zealand, Denmark, need to be modified, and features including dimming control, timer and motion sensor, and the wavelength spectrum should be adjusted to have less blue content.

**Funding:** This research is part of the project “Sustainable Outdoor Lighting for Improved Biodiversity” that has received funding from *We Build Denmark*.

### 2.31. Subjective and Objective Light Sensitivity Under Different Pharmacological Conditions

Lucien Bickerstaff ^1^, Josef Trinkl ^1^, Stephan Munkwitz ^1^, Manuel Spitschan ^2^

^1^  Max Planck Institute for Biological Cybernetics, Tübingen, Germany
^2^  Technical University of Munich, München, Germany

**Abstract:Background**: Light is essential for human physiology and behavior, ranging from visual perception to the non-visual effects of light. The neurobiological pathway underlying all effects of ocular light exposure starts in the photoreceptors in the retina. Prior research has demonstrated that sensitivity to light can differ widely between individuals. There are many ways to operationalize and measure light sensitivity (e.g., pupil constriction, squinting in response to light, visual discomfort ratings, melatonin suppression), yet to date there is no unified framework for light sensitivity across these modalities. In this study, we are characterizing light sensitivity using a variety of subjective and objective methods and across pharmacological manipulations. **Methods**: Healthy volunteers (n = 4, age range 22–47 years) participated in four in-laboratory sessions following a comprehensive screening session. Spectrally calibrated light stimuli were generated using a 10-primary tunable light source (Spectra Tune Lab, Ledmotive Technologies) illuminating a full-field integrating sphere. After a 10 min darkness adaptation period, the participants were presented with cycles of light stimulation: 30 s of white light exposure (0, 450, 1000, 4500, or 18000 lux—counterbalanced) followed by 30 s of refractory darkness in a counterbalanced sequence. Throughout the experiment, EMG of the orbicularis oculi were recorded (to capture blinking, squinting, and sneezing) and pupillometry data were collected. After each stimulus, participants reported their discomfort levels on a 7-point scale verbally. This protocol was performed under four pharmacological conditions delivered through eye drops (one for each visit randomized in order). The pharmacological agents used were the following: oxybuprocaine to anesthetize the cornea, tropicamide to dilate the pupil, a 5% NaCl solution to transiently irritate the cornea, and artificial tears as a negative control. The eye drops were administered four times during the trial: once before the darkness period, once before light stimulation, once after the eighth cycle (after ~10 min), and one last time after the sixteenth cycle (after ~20 min). **Results**: Preliminary pilot data on one individual were collected in 2022, revealing a negative relationship between illuminance and pupil size (R2 = 0.900, F(1, 2) = 18.07, *p* = 0.0511), and a positive relationship between illuminance and facial sensations (R2 = 0.966, F(1, 2) = 56.57, *p* = 0.0172). We expect the additional data to confirm these results, and to show an additional positive relationship between illuminance and both squint- and sneeze-related EMG, and visual discomfort. Moreover, we anticipate that our protocol will cause some individuals to sneeze. **Conclusions**: Our protocol has been demonstrated to be feasible, allowing for its deployment at scale. The data will allow for the development of a more nuanced understanding of the subjective and objective determinants of light sensitivity in humans.

**Keywords**: light sensitivity; pupillometry; electromyography (EMG); pharmacology; photic sneezing

### 2.32. Effect of Constant Light on the Rhythmic Expression of Clock Genes and Kynurenine Pathway Enzyme Genes in the Brain of Rat Pups

Aneta Kubištová, Veronika Spišská, Zdeňka Bendová

Department of Physiology, Faculty of Science, Charles University, Prague, Czech Republic

**Abstract:** A synchronized circadian system is essential for the proper functioning of most physiological processes in the body. This system is synchronized mainly by light, specifically by a sufficiently bright day and a sufficiently dark night. In adult rats with a long-term desynchronized circadian system (for example, caused by constant light), insulin resistance develops, the risk of cancer increases, the quality of sleep decreases, and metabolic syndrome develops overall. These animals have also a significantly reduced amplitude of rhythmic expression of clock genes after several weeks of exposure to constant light. Although the changes caused by constant light in adult rats have been studied in great detail, the effect on juvenile rats is still not well understood. In our experiment, we tested the effect of constant light on Long–Evans rat pups for 30 days (P0–P30) and analyzed changes in the expression rhythms of clock genes and the gene expression of kynurenine pathway enzymes in the central nervous system. Using RT-qPCR analysis, we detected an acrophase shift in clock gene rhythms mostly without a significant reduction in amplitude. We also observed significant changes in gene expression of kynurenine pathway enzymes, which could have a wide-ranging effect on the physiology of the central nervous system.

**Keywords**: circadian clock; constant light; kynurenine pathway; development

**Funding:** This work was funded by the Czech Science Foundation (grant no. 23-07184S).

### 2.33. Protocol for Co-Designing an mHealth Behavior Change Intervention to Optimize Light Exposure with Older Adults in Singapore

Zahrah Alkaff ^1,2^, Resshaya Roobini Murukesu ^1,2^, Denz Del Villar ^1,2^, Manuel Spitschan ^2^

^1^  TUMCREATE
^2^  TUM School of Medicine & Health, Department of Health and Sport Sciences, Tech-nical University of Munich, Munich, Germany

**Abstract:Background**: Light is an important environmental factor in healthy aging, yet age-related eye pathologies and lifestyle choices can hinder this trajectory. Reduced behavioral exposure to light compounded with decreased lens transmittance due to aging can disrupt circadian rhythms, sleep–wake cycles, mood, and cognitive function. Integrating mobile health (mHealth) technology with behavior change strategies as a lifestyle-based intervention offers a promising avenue to optimize light exposure. However, engaging older adults with such interventions can be challenging due to low digital confidence and privacy concerns. There are no targeted and co-designed mHealth smartphone-based applications (app) to optimize healthy light exposure behaviors. This study bridges this gap by developing a user-centered mHealth app for older adults and involving them as co-designers. **Objective**: This study aimed to co-design and develop the LightSPAN mHealth app tailored to optimize light exposure among Singapore’s older adults. **Methods:** This study will involve community-dwelling older adults (target n = 20, aged >60 years) who use smartphones, recruited from aging centers in Singapore. Over three months, we will engage participants through telephone interviews, focus group discussions, and prototyping workshops. During the telephone interviews, participants will respond to the General Health Questionnaire (GHQ-12) for mental health, the Flourishing Scale (FS) for psychological well-being, the Physical Activity Scale for the Elderly (PASE) for self-reported physical activity, and the Pittsburgh Sleep Quality Index (PSQI) for sleep quality assessment, and structured interview questions covering light exposure, smartphone use, and mHealth literacy. The focus group discussions will explore participants’ experiences and perceptions of light exposure, motivations for behavior change, smartphone usage, acceptance of mHealth interventions, and their feedback and initial acceptability of the LightSPAN mHealth intervention. A Feature Preference Questionnaire (FPQ) for the app will then be administered. In the prototyping workshops, participants will review storyboards and design mock-ups of the app, engage in a voting activity to select preferred design options and provide feedback on navigational flow, challenges, likes, dislikes, and suggestions for refinement. All collected data will be analyzed comprehensively, with qualitative data undergoing thematic analysis and quantitative data subjected to descriptive analysis. To ensure a comprehensive and evidence-based approach to app development, these insights will be integrated with consultations from community service providers and a thorough literature review on light exposure interventions, behavior change theories, mHealth user personas, and mHealth design recommendations for older adults. **Conclusions**: The present co-design protocol outlines the formative steps of the LightSPAN project, presenting the systematic procedures undertaken to develop a mHealth behavior change intervention to optimize light exposure among Singapore’s older adults. The insights gained will inform the app’s development and guide subsequent steps, including testing and implementation. By incorporating stakeholder feedback and evidence-based insights, this study aspires to create a user-friendly, effective app that meets older adults needs and empowers them with the tools to manage their light exposure effectively, thereby promoting better circadian health, mood, and cognitive function, and overall well-being.

**Funding:** The National Research Foundation Singapore (NRF2022-THE004-0002).

### 2.34. Time-Of-Day Impacts Severity of Chemotherapy-Induced Fatigue in Mice

Anneke Kastelein, Mayke Tersteeg, Laura Kervezee, Jacques Neefjes, Johanna Meijer

LUMC, Leiden, The Netherlands

**Abstract:** Cancer-related fatigue (CRF) is a common and debilitating condition, affecting cancer patients and survivors years after treatment. To date, underlying mechanisms remain unclear. We have previously shown that doxorubicin (Adriamycin) has a particularly detrimental effect on CRF. Whether this effect is dependent on the time of day is currently unknown. In this study, we aimed to investigate whether treatment time of doxorubicin (DOX) has an effect on the expression and underlying pathways of the fatigue phenotype in mice. Sixty-four female C57BL/6J mice were single housed in 12:12 light-dark (LD) conditions. Animals were treated at ZT2, ZT8, ZT14, or ZT20. At each of these time points, the mice received four injections of 5mg/kg DOX (n = 8) or saline (n = 8) over a period of two weeks. Voluntary wheel running activity (VWRA) as well as home-cage activity was recorded, before (baseline), during, and after treatment. The degree of fatigue was assessed by comparing the levels of baseline and post-treatment activity. Furthermore, circadian characteristics including activity profiles and periodogram-derived Qp values were analyzed. Three weeks after final injection, the animals were placed in constant darkness (DD) for three weeks. After this period, the animals were sacrificed and SCN, PVN, heart, liver, spleen, and muscle were collected as well as serum. All DOX-treated animals showed reduced running wheel activity and home-cage activity compared with their baseline and control animals. Preliminary results suggest that animals treated at ZT14 expressed a more severe fatigue profile after DOX treatment compared with ZT2, ZT8, and ZT20. This was evidenced by a higher reduction in VWRA and home-cage activity, which persisted up to six weeks post-treatment when the experiment was ended. In conclusion, administering treatment at the beginning of the active period appears to be more detrimental compared with other times of day. Adjusting the timing of treatment may therefore lead to a significant improvement of the quality of life of cancer patients and survivors.

### 2.35. Estimating the Downstream Perturbative Effects of Light Stimuli Used to Probe the Circadian Modulation of Retinal Function

Salma M. Thalji ^1^, Hannah Sophie Heinrichs ^1^, Manuel Spitschan ^2^

^1^  Max Planck Institute for Biological Cybernetics, Tübingen, Germany
^2^  Technical University of Munich, München, Germany

**Abstract:Background**: Light exposure can delay or advance the circadian clock through light-sensitive pathways in the retina. To measure photoreceptor function in the retina requires light stimuli itself, thereby potentially affecting the circadian clock themselves. Characterizing how photoreceptor function changes across the circadian cycle therefore poses theoretical and practical challenges, as any stimulus that can be used to probe visual function could also affect neuroendocrine and circadian photoreception. We investigated the impact of circadian rhythms on retinal function in a 40 h short forced-desynchrony (FD) study (comprising 2 h 30 m dim light wake and 1 h 15 m no light sleep intervals). Here, we aimed to quantitatively assess the impact of light stimuli necessary to probe retinal function on vigilant attention and sleepiness. **Methods**: In addition to the main FD study, seven healthy participants (4 male and 3 female; age range 19–30 years, mean = 23.0, S.D. = 3.2) also took part in two additional “experimental-control” evening sessions: a five-hour “light exposure” (LE) session in a <10 lux environment with experimental light stimulus assessments, conducted one week into a pre-FD circadian stabilization period, and a four-hour “dim-light” (DL) session in the same environment but without experimental light stimulus assessments, conducted one week later, on the eve of their FD study start. Both sessions were scheduled to begin four hours before the participants’ habitual bedtimes. The experimental light assessments during the LE session were identical to those experienced in a single sleep–wake cycle of the 40 h FD protocol and comprised (1) pupillometric assessments using silent-substitution stimuli delivered in Maxwellian view (approx. 25 min per block; n = 11 blocks) and (2) psychophysical assessments of temporal contrast thresholds (approx. 30 min per block; n = 11 blocks). Throughout the evening, participants completed the visual psychomotor vigilance task (PVT) test sessions every hour, and the Karolinska Sleepiness Scale (KSS) every half hour. **Results**: Qualitative comparison of PVT performance metrics and KSS scores between the LE and DL sessions suggests a slight decrease in both objective and subjective measures of vigilant attention during the LE session (population mean ± SEM: median reaction time (RT) 251.80 ± 5.03 ms vs. 262.57 ± 7.45 ms, mean fastest 10%RT 198.72 ± 3.63 ms vs. 200.75 ± 4.28 ms, mean slowest 10%RT 415.51 ± 13.99 ms vs. 433.53 ± 18.44 ms, lapse (RT > 500 ms) count: 1.5 ± 0.32 vs. 2.5 ± 0.61, and KSS score: 5.79 ± 0.25 vs. 5.84 ± 0.24). This observation aligns with our hypothesis that light stimuli during the protocol may modify the circadian phase of alertness. However, statistical analysis using non-parametric tests (Wilcoxon signed-rank test for each of the time points (PVT n = 4, KSS n = 9), with Bonferroni correction for multiple testing) did not reveal any significant differences (*p* > 0.05) between the two experimental conditions. These tests were underpowered, which may have affected the detection of significant effects. **Conclusions**: Our results provide suggestive evidence that using light stimuli to probe retinal function may slightly perturb vigilant attention and alertness, thereby potentially challenging the possibility of characterizing non-visual photoreception without leading to downstream perturbations.

**Keywords**: light exposure; circadian rhythms; vigilance

### 2.36. Light Therapy Reduces Daily Intrusive Cognition in Young Adults with Obsessive–Compulsive Disorder (OCD): Preliminary Findings from a Pilot Randomized Control Trial

Rebecca Cox, Alyssa Week, Lidia Ballell, Sophia Haller, Isabella Macarelli, Kenneth Wright

University of Colorado Boulder, Boulder, CO, USA

**Abstract:Background**: Obsessive–compulsive disorder (OCD) is a psychiatric disorder characterized by intrusive, distressing cognition (i.e., obsessions) and repetitive behaviors (i.e., compulsions). Despite established first-line treatments, only 40% of those treated achieve remission, suggesting the presence of untreated mechanistic factors. There is evidence for circadian delay in OCD, including later chronotype, later circadian melatonin phase, and high comorbidity with delayed sleep–wake phase disorder. Thus, delayed circadian rhythms may represent a novel treatment target. Recent studies have shown that bright light therapy is effective for disorders that are phenomenologically similar to OCD, including Tourette’s disorder and posttraumatic stress disorder. We hypothesized that light therapy (including morning bright light and evening dim light) would improve symptoms of OCD. **Methods**: The target sample size for this study was *N* = 30 (15 per treatment condition). This preliminary analysis included *n* = 14 (10 female) young adults (mean_age_ = 21.29 ± 3.27) with OCD and late bedtimes (01:00 or later) who were randomly assigned to 3 weeks of active or placebo light therapy. Participants in the active condition kept a fixed wake time, completed 1 h of morning bright light delivered via wearable light therapy glasses (mean melanopic EDI lux = 570.11 ± 76.87), and were in dim light for 2 h prior to bedtime. Participants in the placebo condition completed 1 h of morning light delivered via placebo glasses (mean melanopic EDI lux = 12.98 ± 7.26) and practiced good sleep hygiene. Participants completed a retrospective interview of OCD symptom severity before and after treatment and monitored sleep and OCD symptoms for 2 weeks before treatment and in the last treatment week via actigraphy and self-report symptom measures administered 4 times/day. Given the small and incomplete sample, we calculated effect sizes (Cohen’s *d*). **Results**: There was a large reduction in daily intrusive cognition from pre- to post-treatment in the active condition (*d* = 1.17) and a small reduction in the placebo condition (*d* = 0.18). There was a medium reduction in daily OCD symptoms from pre- to post-treatment in the active condition (*d* = 0.55) and a small reduction in the placebo condition (*d* = 0.32). There were medium reductions on the retrospective OCD symptom severity interview from pre- to post-treatment in both the active and placebo treatment conditions (*d* = 0.42 and *d* = 0.48, respectively). There was a medium shift towards earlier sleep timing from pre- to post-treatment in the active condition (*d* = 0.62) and a small shift earlier in the placebo condition (*d* = 0.13). **Conclusions**: Participants who received active light therapy reported large and medium reductions in daily intrusive cognition and OCD symptoms, whereas participants in both treatment conditions reported medium reductions in retrospective OCD symptom severity. Three weeks of light therapy may therefore be sufficient to improve symptoms at the daily level but insufficient to detect effects on measures that contain retrospective bias. The medium effect of active light therapy on sleep timing suggests treatment effects observed at the daily level may occur through circadian mechanisms. Future analyses will examine a change in circadian melatonin phase in the complete sample.

**Keywords**: OCD; light therapy; sleep timing

**Funding:** American Academy of Sleep Medicine Foundation 270-FP-22; International OCD Foundation.

### 2.37. Evaluation of the Impact of Electrochromic Glazing on Visual and Non-Visual Light Effects in Office Spaces: A Simulation Comparison with Traditional Glazing and External Blinds in Bratislava

Tomáš Hakszer, Peter Hanuliak

Department of Building Structures, Faculty of Civil Engineering, Slovak University of Technology, Bratislava, Slovakia

**Abstract:Background**: Electrochromic glazing is an innovative technology in the field of adaptive facades, which allows for dynamic regulation of daylight in building interiors. Regulation is possible thanks to the physical principle of electrochromism. The change in the optical properties of the glass from clear to colored darkened glass works on the principle of ion migration between suitable active layers in the presence of an electric voltage. This dynamic change goes beyond simply altering the overall light transmission. It actively modifies the spectral transmittance of the glass, typically resulting in a blue coloration when darkened. This shift in the light spectrum has a significant impact on the visual experience and potentially influences non-visual responses like circadian rhythms. **Methods**: Our study employed computer simulations to compare the effects of electrochromic glazing and traditional insulation glazing with external blinds on visual and non-visual light in an office space within an administrative building located in Bratislava. The software Climate Studio was utilized to conduct year-round simulations of daylight and its associated visual effects. Based on the outcomes of these year-round simulations, specific times throughout a single day were selected for evaluating melanopic daylight illuminance and melanopic/photopic illuminance ratios using the software ALFA. **Results**: Electrochromic glazing demonstrated the ability to maintain comfortable visual conditions in the workspace, achieve uniform daylight distribution throughout the space, and align with human circadian rhythms. Unlike other shading techniques, it can also facilitate continuous visual connection with the exterior environment. However, complete darkening of the entire glazing area proved insufficient to meet the required daylight illuminance and melanopic daylight illuminance levels. **Conclusions**: Electrochromic glazing shows promise for optimizing office light (visual and non-visual) while maintaining a connection to the outdoors. However, fully darkened states struggle to meet requirements for proper lighting. Future research should investigate maintaining a neutral light spectrum during partial darkening to unlock the full potential of electrochromic glazing for both comfort and circadian health. This could be achieved by dynamically positioning clear patches within the electrochromic glazing, allowing them to adjust the system transparency based on outdoor lighting conditions.

**Keywords**: indoor comfort; daylighting; well-being; advanced glazing

**Funding:** This research was supported by the Slovak Scientific Grant Agency (VEGA) under Grant number 1/0042/21, Slovak Research and Development Agency (APVV) under Grant number 18-0174 and Young STU researchers support program under Grant number 1615.

### 2.38. Chronotherapy with Amlodipine/Lisinopril Combination in Hypertensive Patients

Yklym Bolmammedov, Taganmyrat Hojageldiyev

M.Garryyev State Medical University of Turkmenistan, Aşgabat, Turkmenistan

**Abstract:Background**: Despite the multitude of treatment options for the management of hypertension, cardiovascular complications remain the leading causes of death attributed in part to high blood pressure (BP). In addition to the circadian BP changes, the pharmacodynamics of antihypertensive medications can vary according to the time of administration. Traditional treatment regimens need to be improved based on the principles of chronobiology. **Methods**: A total 96 patients (52 female) with uncomplicated essential hypertension, aged between 42 and 60, were included in this study. This study compared the effects of the amlodipine/lisinopril combination on 24 h blood pressure in groups of hypertensive patients: morning dosing in morning (MM, n = 22) and evening (ME, n = 22) chronotype patients, and evening dosing in both chronotypes (EM, n = 26; EE, n = 26), respectively. Ambulatory blood pressure monitoring (ABPM) was performed before and after therapy, and chronotypes were assessed by the questionnaire in all patients. **Results**: We found that morning and evening dosing of amlodipine/lisinopril had equivalent effects on diastolic BP in both chronotype groups. There was a significant difference in nighttime systolic BP in groups EM and ME (*p* < 0.05), and a slight but not significant difference in both nighttime and daytime BP between groups MM and EE. **Conclusions**: The results show that morning dosing of amlodipine/lisinopril is preferred in evening chronotypes, while evening dosing is better in morning chronotypes for optimal management of hypertension. More detailed studies will help to achieve the greatest effectiveness of the chronotherapy of essential hypertension and decrease negative outcomes of this disease.

### 2.39. Patients with Autoimmune Adrenal Insufficiency Exhibit Differing Chronotype, Sleep Onset, and Psychology-Based Task Performance in Comparison with Healthy Controls

Lakshmi Kalathinkunnath ^1^, Kristin Tessmar-Raible ^1^, Helmut Leder ^1^, Peter Wolf ^2^, Till Roenneberg ^3^, Fani Sentinella Jerbic ^4^, Jozsef Arato ^1^, Michael Krebs ^2^, Hannes Beiglböck ^2^

^1^  University of Vienna, Wien, Austria
^2^  Medical University of Vienna, Wien, Austria
^3^  Institute of Medical Psychology, LMU Munich, Munich, Germany
^4^  Technical University of Vienna, Vienna, Austria

**Abstract:Background**: Human chronotypes are a consequence of the circadian clock individual periods and are observed to have a significant impact on cognitive performance. Cortisol is a key mediator for the rhythmic expression of circadian signals in almost all tissues, physiological timing of which is strictly regulated by the hypothalamic–pituitary–adrenal (HPA) axis. Diseases affecting HPA signaling might therefore have important consequences on the individual’s chronotype, and hence their performance in various psychology-based tasks. We therefore investigated primary adrenal insufficiency patients with stable hydrocortisone replacement therapy (medicine self-administered between wake-up time and 2 PM) and a healthy control population by monitoring their chronotype, sleep parameters, and performance in various psychology-based tests. **Methods**: In this exploratory pilot study, 22 patients with autoimmune adrenalitis and 40 control subjects of comparable age (53.77 ± 17.27 vs. 47.77 ± 14.94 years) and sex (15 f/7 m vs. 31 f/9 m) were assessed for their chronotypes using the Munich Chronotype Questionnaire and subjected to five different psychology-based tasks at least four times. They were also requested to constantly wear an actimetry device for 12 weeks that monitored their sleep, temperature, and light reception. F-tests were applied to test for variability in sleep parameters and chronotypes between patients and controls, and a one-way ANOVA was applied to test for significant differences between performance measures for the different tasks, and between means of sleep onset of the two cohorts. **Results**: We observed high variability in sleep onset (F statistic =1.876, *p* < 0.001) and sleep duration (F statistic = 2.496, *p* < 0.001) for patients in comparison with the controls. The mean value for sleep onset were also observed to be earlier for patients (mean = 131.708 min) in comparison with the controls (mean = 155.803 min) (confirmed with one way ANOVA, statistic = 29.842, *p* < 0.001). Lower performance was observed for patients in comparison with the controls of the earlier chronotype, in tests that measured attention (*p* < 0.05) and aesthetic sensitivity (*p* < 0.05). Chronotypes of patients showed high variability in comparison with the controls (F statistic = 2.192, *p* = 0.008). The patient population also seemed to show a numerically later sleep onset on the weekends, which was not compensated by increased duration of sleep on the next day. **Conclusions**: The changes in chronotype and sleep onset in the patient cohort were likely caused by glucocorticoid replacement and possibly additional hormonal alterations. Lower performance in attention and aesthetic sensitivity for early chronotypes in this cohort may point to the possibility of mild cognitive deficits due to the disease or time of administration of the drug. Improved awareness of their sleep variability and more controlled sleep/wake schedules, as well as illumination, can likely improve the patients’ life quality. Possibly associated clinically relevant effects on cardiovascular risk factors remain to be investigated in future experiments.

**Keywords**: chronotype; primary adrenal insufficiency; sleep onset; actimetry; cognition; mental ability

**Funding:** This work was funded by the Vienna Doctoral School for Cognition, Behaviour and Neuroscience, the University of Vienna, and SFB 78.

### 2.40. The Impact of Age and Lifelong Endurance Training on Sleep Quality, Sleep/Wake Rhythm Stability, and 6-Sulfatoxymelatonin

Katarína Stebelová ^1^, Genc Berisha ^2^, Katarina Kovacova ^1^, Michal Zeman ^1^, Milan Sedliak ^2^, Monika Okuliarova ^1^

^1^  Department of Animal Physiology and Ethology, Faculty of Natural Sciences, Comenius University in Bratislava, Bratislava, Slovakia
^2^  Department of Biological and Medical Sciences, Faculty of Physical Education and Sport, Comenius University in Bratislava, Bratislava, Slovakia

**Abstract:Background**: Lifelong endurance exercise effectively reduces age-related decline in physiological functions and promotes overall health and well-being in older adults. Regular physical activity can stabilize the central circadian pacemaker in suprachiasmatic nuclei to the external photoperiod. Our study aimed to find out whether lifelong endurance running is beneficial for circadian rhythmicity evaluated through sleep/wake rhythms and 6-sulfatoxymelatonin. **Methods**: In this study, 38 men were analyzed. They were divided into four groups: young endurance runners (YA, n = 10, 27.9 ± 0.9 yrs.), young sedentary (YS, n = 8, 25.8 ± 0.8 yrs.), master endurance runners (MA, n = 11, 69.5 ± 1.4 yrs.), and elderly sedentary (ES, n = 9 71.3 ± 1.3 yrs.). Active groups (YA, MA) included professional runners who run more than 300 min per week and actively participate in competitions from 10 km to the marathon. In sedentary groups (YS, ES), there were individuals with no history of regular physical training. Sleep/wake rhythm was monitored for one week using wrist actigraphy (CamNtech, UK). The sleep parameters, intra-daily variability (IV), and inter-daily stability (IS) were evaluated using the software MotionWare 1.3.17 (CamNTech, UK). IV quantified the degree of fragmentation of activity–rest periods and IS quantified the degree of regularity in the activity–rest pattern. Concentrations of 6-sulfatoxymelatonin were measured in evening and morning urine using ELISA (DRG, Germany). Mesor, amplitude, and acrophase were calculated via cosinor analysis. **Results**: Young and elderly sedentary men had significantly higher body weight, body fat, and BMI compared with age-matched endurance runners. As expected, YA and MA had higher mesor and amplitude of daily activity rhythms. The acrophase did not differ among groups. YA had the highest activity among all participants but also significantly lower sleep efficiency, higher fragmentation index, lower actual sleep, and higher actual wake compared with YS. Elderly participants slept longer and had higher sleep efficiency than young participants regardless of activity status. MA had longer immobile time during sleep compared with ES. Higher morning 6-sulfatoxymelatonin/creatinine values were found in YA and MA compared with age-matched controls. Parameters reflecting the stability and variability of the circadian system (IV and IS) were significantly different in YA compared with all other groups. YA had more variable and less stable daily rhythms. **Conclusions**: Young and elderly lifelong endurance athletes had significantly higher morning urinary 6-sulfatoxymelatonin, the established marker of effective entrainment. On the other hand, the worst sleep quality, and altered IV and IS in young athletes can reflect their disturbed daily regimes reflecting their highly variable lifestyle.

**Keywords**: aging; 6-sulfatoxymelatonin; sleep quality; intra-daily variability; inter-daily stability

**Funding:** Grant no. APVV-21-0164.

### 2.41. Reduced Intensity and Light Spectrum Impact on Salivary Hormones and Sleep Quality in Office Workers

Katarina Kovacova ^1^, Zuzana Dzirbikova ^1^, Peter Hanuliak ^2^, Peter Hartman ^2^, Andrea Vargova ^2^, Jozef Hraška ^2^, Katarína Stebelová ^1^

^1^  Department of Animal Physiology and Ethology, Faculty of Natural Sciences, Comenius University in Bratislava, Bratislava, Slovakia
^2^  Department of Building Structures, Faculty of Civil Engineering, Slovak University of Technology, Bratislava, Slovakia

**Abstract:Background**: The circadian system allows the adaptation of organisms to external light-dark cycles. Light exposure, particularly in the morning hours, acts as the primary entraining factor for the human circadian system, promoting organism activation. Conversely, darkness during the night stimulates the production of the hormone melatonin. Melatonin, cortisol, and sleep–wake rhythms are prominent outputs directly regulated by the circadian system. This study investigated the effects of reduced short-wavelength light exposure during the day on salivary melatonin, cortisol levels, and sleep–wake patterns in both women and men. **Methods**: Twenty-three healthy adults (15 females, 8 males; mean age ± SD: 26.3 ± 1.6 years) participated in this study. This study was conducted for 14 consecutive days during the winter months. This study took place in two structurally identical office environments. The reference office received natural daylight, while the experimental office had windows and light fixtures covered with a spectrally tuned foil that transmitted only 3.6% of light wavelengths up to 500 nm. Participants provided every morning (upon waking) and evening (before sleep) saliva samples for hormone analysis (melatonin, cortisol, ELISA). Participants self-reported their sleepiness using the Karolinska Sleepiness Scale in the morning and evening. They wore light dataloggers (Object Tracker, Austria) throughout the study to objectively monitor light intensity and spectral composition. Wrist actigraphy using MotionWatch 8 (CamNTech, UK) was used to assess sleep–wake patterns and sleep quality. **Results**: Men, but not women, had significantly higher sleepiness during the experimental week compared with the reference week. On the other hand, women had significantly worse sleep quality during the experimental week, while men did not show differences in sleep quality. The concentration of salivary melatonin did not differ in the evening or morning due to the reduced light spectrum. As expected, the morning cortisol concentration significantly differed between sexes with higher levels in women compared with men but not due to light modification. **Conclusions**: Our results suggest that reduced blue light and intensity during the day have no direct impact on salivary melatonin and cortisol concentration. Worsened objective sleep quality in women and interestingly, greater subjective sleepiness in males point out intersexual differences. These findings indicate sex-dependent reactions to lighting conditions and warrant further investigation.

**Keywords**: melatonin; cortisol; sleep; blue-light reduction; daylight

**Funding:** This study was supported by grant APVV-18-0174, APVV-21-0223, UK/1161/2024, and the Operation Program of Integrated Infrastructure for the project, Advancing University Capacity and Competence in Research, Development and Innovation, ITMS2014+: 313021 × 329, co-financed by the European Regional Development Fund.

### 2.42. Influence of Light Direction (mEDI) and Spectrum (mDER) on Melatonin Suppression and Measures of Alertness in the Workplace

Kai Broszio ^1^, Martine Knoop ^2^, Ljiljana Udovicic ^1^, Anne Helene Garde ^3^, Stephan Völker ^2^

^1^  Federal Institute for Occupational Safety and Health (BAuA), Dortmund, Germany
^2^  TU Berlin, Berlin, Germany
^3^  The National Research Centre for the Working Environment, Copenhagen, Denmark

**Abstract:Background**: Night shift work is on the rise in modern society. It is associated with exposure to light at night (LAN) and with health problems. Melatonin suppression may be a risk factor. Therefore, strategies to reduce melatonin suppression while maintaining alertness are needed. One lever might be spectral tuning, because low mDER light has been reported to support alertness while having little or no effect on melatonin concentration and phase shift. Another leverage might be spatial tuning, because light from the superior field of view (FOV) is reported to suppress melatonin more than light from the inferior FOV. The research question is as follows: Is it possible to minimize melatonin suppression and at the same time support alertness for night work by designing spatially and spectrally optimized lighting? The hypotheses are the following: (1) Lighting conditions with mEDI 200 lx (A1 and A2) suppress melatonin significantly more and increase measures of alertness significantly more than lighting conditions with mEDI 100 lx (B). (2) Lighting conditions (A1) with 80% of the mEDI coming from the superior FOV suppress significantly more melatonin and increase measures of alertness than lighting conditions (A2) with 80% of the mEDI coming from the inferior FOV at the same full FOV mEDI and the same full FOV illuminance. The latter may be especially interesting for professions with a high percentage of fixed viewing directions, e.g., control room workers, flight controllers, etc. **Methods**: A complete counterbalanced crossover repeated measures within-subjects laboratory study was conducted. A total of 36 (f = 18) participants (mean age 27a, SD 4a) participated in 3 laboratory sessions of 5 h each, each consisting of 1 h dim followed by 3 h exposure followed by 1 h dim. Inclusion criteria were general health (SF-12), chronotype (MEQ and MCTQ), depression (PHQ), and sleep quality (PSQI) in the normal range. There were 3 different lighting settings with fixed viewing direction: A1: mEDI 200 lx with 80% of the mEDI coming from the superior FOV, total mDER 1.43. A2: mEDI 200 lx with 80% of the mEDI coming from the inferior FOV, total mDER 1.43. B: mEDI 100 lx with 50% of the mEDI coming from either inferior or superior FOV, total mDER 0.48. Ambient air temperature, relative humidity, and carbon dioxide were recorded and controlled by an HVAC system. Dependent variables included melatonin (saliva samples; liquid chromatography-tandem mass spectrometry) and measures of alertness (auditory versions of the PVT, Go/NoGo and n-back, and the KSS). **Results**: Data were preprocessed and melatonin data were log-transformed. To assess hypothesis 1, we applied a linear mixed model with time of measurement and light condition as fixed factors, subject as a random factor, and light condition as a random slope. We found that melatonin suppression was significantly higher for mEDI 200 lx (A1 and A2) compared with mEDI 100 lx (B) for all measurement times during exposure. Measures of alertness did not show a statistically significant difference, but for most cognitive measures, mean reaction times were faster under mEDI 100 lx (B). The same LMM was used for hypothesis 2. Melatonin suppression was higher in the condition with 80% mEDI from the superior FOV at all measurement times but reached a significant difference only after 3 h of exposure. All measures of alertness showed no significant difference, but PVT mean response times were significantly faster for all measurement times under 80% mEDI from the inferior FOV. **Conclusions**: By tuning to more reddish light (mDER 1.43 to 0.48), melatonin suppression was significantly reduced, while alertness was not significantly reduced. Blueish light from the upper FOV suppressed melatonin (sig. after 3 h exposure) and did not significantly influence alertness (except for PVT). Thus, the results suggest that it might be possible to improve the night shift workplace by optimizing the spectral and spatial lighting conditions. In general, it might be good to light night shift workplaces with low mDER light and if there is high mDER light, it should not come from the upper FOV.

### 2.43. Virtual Darkness for Agitation in Dementia: The DARK.DEM Randomized Controlled Trial

Sunniva Vibe Skagen ^1^, Tone Elise Henriksen ^2^, Stein Erik Fæø ^3^, Kjersti Nedreskår ^1^, Elisabeth Flo-Groeneboom ^4^, Line Iden Berge ^1^

^1^  Centre for Elderly and Nursing Home Medicine, University of Bergen, Bergen, Norway
^2^  Valen Hospital, Department of Research and Innovation, Fonna Health Trust, Haugesund, Norway
^3^  VID Specialized University, Oslo, Norway
^4^  Department of Clinical Psychology, University of Bergen, Bergen, Norway

**Abstract:Background**: Behavioral and psychological symptoms (BPSDs) such as depression, anxiety, disrupted sleep, and agitation, are prevalent in people with dementia [1]. BPSDs are associated with reduced cognitive and physical functioning, reduced quality of life, increased risk of institutionalization, and increased mortality [1,2]. Pharmacological treatment of BPSDs is demanding with limited efficacy and severe side-effects [1,3,4]. People with dementia often have an altered rest–activity rhythms, which again potentiates BPSDs [1,5]. Various chronotherapeutic approaches, i.e., interventions targeting regulation of activation and the circadian rhythm, have been explored to compensate for destabilized rest–activity rhythm in people with dementia. The cluster randomized DEM.LIGHT trial found some improvement in depression and sleep after 24 weeks of bright light therapy (BLT) in people with dementia in nursing homes [6,14]. It might be that BLT has less effect in older adults due to limited light reaching the retina as a result of eye conditions. Virtual darkness therapy (VDT) aims at regulating activation and the circadian rhythm by restricting short wave-length light during the evening and night [7]. Studies have found VDT effective in reducing manic symptoms in bipolar disorder [8] and for improving sleep and circadian function in healthy adults [9]. DARK.DEM aims to develop and evaluate VDT to enhance treatment of BPSDs in dementia care. **Methods:** The DARK.DEM trial will be conducted at NKS Olaviken Gerontopsychiatric Hospital. A total of 72 patients will be randomized to either treatment as usual or 14 days of blue wavelength depleted evening light provided with circadian lighting in secluded units from 19.00 to 08.00. Inclusion criteria: dementia diagnosis, all stages and etiologies, ≥50 years, both genders, and clinically significant agitation (Cohen-Mansfield Agitation Scale, CMAI ≥ 45 [10]). Exclusion criteria: total blindness/diminished bilateral red reflex, use of beta-blockers and/or melatonin, and clinically significant pain (MOBID-2 ≥ 3) [11]. The intervention will be provided as an add-on treatment to treatment as usual, which includes use of psychotropic drugs, environmental interventions, music therapy, and use of medical restraints. Data will be collected at four time points: baseline, day 7, day 14, and at discharge. The study will start in August 2024 and run for 30 months. The DARK.DEM trial has been approved by the Regional Committees for Medical and Health Research Ethics (ref. nr. 697405). **Expected Results**: The primary outcome is a change in sum score of agitation (CMAI). Secondary outcomes are a change in neuropsychiatric symptoms (The Neuropsychiatric Inventory [12], Cornell Scale for Depression in Dementia [13]), quality of life, activities of daily living, use of psychotropic drugs and restraints, and length of hospital stay.

**Keywords**: chronotherapy; darkness therapy; dementia; agitation; BPSD

**Funding:** The DARK.DEM project is funded by The Research Council of Norway and the University of Bergen.

**References**

1. Vik-Mo, A.O.; Giil, L.M.; Borda, M.G.; Ballard, C.; Aarsland, D. The individual course of neuropsychiatric symptoms in people with Alzheimer’s and Lewy body dementia: 12-year longitudinal cohort study. *Br. J. Psychiatry* **2020**, *216*, 43–48.
2. World Health Organization, Fact Sheet Dementia. 2021. Available online: https://www.who.int/news-room/fact-sheets/detail/dementia.
3. Bessey, L.J.; Walaszek, A. Management of Behavioral and Psychological Symptoms of Dementia. *Curr. Psychiatry Rep.* **2019**, *21*, 66.
4. Watt, J.A.; Goodarzi, Z.; Veroniki, A.A.; Nincic, V.; Khan, P.A.; Ghassemi, M.; Thompson, Y.; Lai, Y.; Treister, V.; Tricco, A.C.; et al. Safety of pharmacologic interventions for neuropsychiatric symptoms in dementia: a systematic review and network meta-analysis. *BMC Geriatr.* **2020**, *20*, 212.
5. Logan, R.W.; McClung, C.A. Rhythms of life: circadian disruption and brain disorders across the lifespan. *Nat. Rev. Neurosci.* **2019**, *20*, 49–65.
6. Hjetland, G.J.; Kolberg, E.; Pallesen, S.; Thun, E.; Nordhus, I.H.; Bjorvatn, B.; Flo-Groeneboom, E. Ambient bright light treatment improved proxy-rated sleep but not sleep measured by actigraphy in nursing home patients with dementia: a placebo-controlled randomised trial. *BMC Geriatr.* **2021**, *21*, 312
7. Scott, J.; Langsrud, K.; Goulding, I.R.; Kallestad, H. Let there be blue-depleted light: in-patient dark therapy, circadian rhythms and length of stay. *BJPsych Adv.* **2020**, *27*, 73–84.
8. Henriksen, T.E.; Skrede, S.; Fasmer, O.B.; Schoeyen, H.; Leskauskaite, I.; Bjørke-Bertheussen, J.; Assmus, J.; Hamre, B.; Grønli, J.; Lund, A. Blue-blocking glasses as additive treatment for mania: a randomized placebo-controlled trial. *Bipolar Disord.* **2016**, *18*, 221–232.
9. Vethe, D.; Scott, J.; Engstrøm, M.; Salvesen, Ø.; Sand, T.; Olsen, A.; Morken, G.; Heglum, H.S.; Kjørstad, K.; Faaland, P.M.; et al., The evening light environment in hospitals can be designed to produce less disruptive effects on the circadian system and improve sleep. *Sleep* **2021**, *44*, zsaa194.
10. Rabinowitz, J.; Davidson, M.; De Deyn, P.P.; Katz, I.; Brodaty, H.; Cohen-Mansfield, J. Factor analysis of the Cohen-Mansfield Agitation Inventory in three large samples of nursing home patients with dementia and behavioral disturbance. *Am. J. Geriatr. Psychiatry* **2005**, *13*, 991–998.
11. Husebo, B.S.; Ostelo, R.W.J.G.; Strand, L.I. The MOBID-2 pain scale: reliability and responsiveness to pain in patients with dementia. *Eur. J. Pain* **2014**, *18*, 1419–1430.
12. Cummings, J. The Neuropsychiatric Inventory: Development and Applications. *J. Geriatr. Psychiatry Neurol.* **2020**, *33*, 73–84.
13. Alexopoulos, G.S.; Abrams, R.C.; Young, R.C.; Shamoian, C.A. Cornell Scale for Depression in Dementia. *Biol. Psychiatry* **1988**, *23*, 271–284.
14. Kolberg, E.; Hjetland, G.J.; Thun, E.; Pallesen, S.; Nordhus, I.H.; Husebo, B.S.; Flo-Groeneboom, E. The effects of bright light treatment on affective symptoms in people with dementia: A 24-week cluster randomized controlled trial. *BMC Psychiatry* **2021**, *21*, 377.

### 2.44. Examining Time-of-Day Variation in Human Temporal Contrast Sensitivity in an Ultra-Short Sleep–Wake Protocol

Hannah Sophie Heinrichs ^1^, Salma M. Thalji ^1^, Manuel Spitschan ^2^

^1^  Max Planck Institute for Biological Cybernetics, Tübingen, Germany
^2^  Technical University of Munich, München, Germany

**Abstract:Background**: Many physiological and psychological functions, including the sensitivity of the retina, vary throughout the day due to being governed by the central circadian clock. Studies in mice suggest that retinal tissue and, specifically, photoreceptor signaling, exhibit a diurnal rhythm. Previous studies in humans have investigated variability in luminance and color sensitivity but have not characterized this variability at the level of the post-receptoral luminance and color channels (achromatic, L+M+S; red-green, L-M; blue-yellow, S-[L+M]). To target these post-receptoral mechanisms in isolation, the method of silent substitution is necessary. Here, we investigate circadian or time-of-day variations in human temporal contrast sensitivity for color and luminance. We probe human performance using repeated psychophysical experiments with temporally defined and spatially homogenous stimuli over 40 h. We expected to confirm that contrast sensitivity for high-frequency (8 Hz) compared with low-frequency (2 Hz) modulation will differ based on the targeted retinal mechanism. **Methods**: Healthy volunteers (n = 6, 21.83 ± 1.57 years, 50% female) participated in a 40 h experimental protocol comprising 11 experimental blocks that were interspersed with sleep opportunities yielding a sleep–wake ratio of 1:2 (2:30 h sleep and 1:15 h wakefulness). During each experimental block, participants completed three different psychophysical detection tasks targeting the luminance channel (L+M+S), the red-green channel (L-M), and the blue-yellow color channel (S-[L+M]) on a calibrated monitor system (Metropsis Research, Cambridge Research Systems) viewed by the participant’s eye through a pupil relay system. The 2AFC tasked each probe the temporal contrast sensitivity of retinal mechanisms at two frequencies, 2 Hz and 8 Hz, and they were repeated three times per block. A staircase method was used on the stimulus’ contrast levels to find the detection threshold. **Results**: Our preliminary data of the temporal contrast sensitivity of different post-receptoral channels indicate distinct differences in average contrast sensitivity depending on post-receptoral channel and temporal frequency. For both 2 and 8 Hz, temporal sensitivity for the red-green (L-M) channel was highest. At 8 Hz, the temporal sensitivity for the luminance channel (L+M+S) was higher than at 2 Hz, indicating bandpass response for the luminance channel. The present dataset does not show a clear, discernible diurnal or circadian rhythm across repeated measurement sessions. **Discussion**: Our results are consistent with the literature, displaying higher sensitivity to red-green (L-M) stimuli compared with luminance (L+M+S) and blue-yellow (S-[L+M]) at low temporal frequencies and temporal bandpass properties in the luminance channel. This is the first study to use psychophysical test stimuli based on carefully modulating post-receptoral mechanisms repeatedly over 40 h. Future analysis will include inferential statistics on diurnal or circadian rhythmicity by fitting a linear mixed model.

### 2.45. The Photon Space: Study Protocol and Preliminary Data from Daylight Research in Northern Sweden

Kateřina Červená ^1^, Soufien Sghari ^1^, Wayne Iwan Lee Davies ^2^, Katharina Wulff ^1^

^1^  Department of Molecular Biology, Umeå University, Umeå, Sweden
^2^  Department of Medical and Translational Biology, Umeå University, Umeå, Sweden

**Abstract:Background**: Daylight was used for centuries to prevent and cure human diseases. But it has dropped out in modern times despite its enormous benefits for a healthy brain. Today, electrical light interacts and competes with the natural light, and studying people purely under the influence of daylight all year round requires unique infrastructures. **Methods and Results**: The Nordic Daylight Research Facility, so-called “Photon Space”, is such a unique, all-glass pavilion, thermally well insulated and located just 2.3° south of the Arctic circle. Its secluded location protects it from artificial light pollution and positioned at high seasonality which allows us to study its effects on human physiology and behavior over a wide range of natural light qualities throughout changing seasons. Apart from transmitting natural light, the Photon Space is equipped with controllable electrical lightning to compare the effects of artificial light of different parameters on retinal sensitivity, sleep, mood, and subjective experience. We present our protocol together with preliminary data from the indoor habitat and our participants. In this 2.5-week-long study protocol the participant sleep/activity rhythm and light exposure was recorded continuously using actigraphy. After one week of baseline recording in their home setting the participant spent 72 h in the Photon Space, where multimodal data were collected repeatedly during changing natural light, from dawn to dusk and in between, as well as under pre-selected and self-selected electrical light. Subjective longitudinal data using scales and digital ecological momentary sampling, along with wearable devices, pupillometry, eye tracking, and brain activity were part of physiological and behavioral monitoring. The indoor milieu conditions were captured using a variety of sensors including a photo-spectrometer and multi-sensor recorders to capture illuminance, UV, humidity, and temperature. **Conclusions**: The Photon Space aims to close an infrastructure gap to provide new knowledge on basic questions related to sleep and mood regulation by daylight. It is expected to help reevaluate and adapt lighting regimes for the Nordic habitats with long twilight phases to be accounted for in local architecture and lighting designs.

**Keywords**: daylight; twilight; photoperiod; sleep; subjective experience

### 2.46. Hot or Cold: Skin Temperature as a Predictor of Daytime Sleepiness?

Vaida Verhoef ^1^, Karin Smolders ^1^, Geert Peeters ^2^, Sebastiaan Overeem ^2^, Yvonne de Kort ^1^

^1^  Human Technology Interaction group, department of Industrial Engineering and Innovation Sciences, Eindhoven University of Technology, North Brabant, The Netherlands
^2^  Kempenhaeghe Sleep Center, Heeze, The Netherlands

**Abstract:Background**: Daytime sleepiness is a common experience among individuals with obstructive sleep apnea (OSA), often underestimated due to challenges in accurate assessment. In pursuit of reliable and sensitive measures, research has shifted from subjective scales to physiological predictors such as skin temperature. Minimally obtrusive, skin temperature is an interesting index of circadian rhythm, whose disruption has recently been linked to the severity of daytime sleepiness in patients with sleep apnea. Building on this hypothesis, this study aimed to explore the extent to which skin temperature relates to momentary experiences of sleepiness in real-life settings. **Methods**: We conducted a randomized crossover study with 17 healthy participants (aged 19–32 years, 7 females, 10 males, PSQI ≤ 5). After a week of baseline, participants underwent a sleep restriction paradigm with two counterbalanced sleep conditions (restricted: 4 h/night vs. normal: 7–9 h/night), each lasting three consecutive nights with a four-night washout period. During each condition, distal (hands) and proximal (clavicle) skin temperatures were continuously recorded (4 iButtons, 1/300 s sampling frequency). Additionally, participants answered repeated momentary sleepiness (KSS, SSS) and fatigue (VAS) assessments throughout the day using the experience sampling method. Skin temperature metrics were analyzed using linear mixed models, with participant ID as a random intercept and sleep condition, day, and time of day as fixed factors (including interaction terms). Relationships between skin temperature (averaged across the hour prior to each questionnaire) and subsequent sleepiness assessments were investigated with linear mixed models in subdivided data (each sleep condition separately). Indices of rhythm were derived from the continuous data using cosinor modeling. **Results**: Distal (DST) and proximal (PST) skin temperatures, as well as their gradient (DPG), were significantly affected by the interaction between sleep condition and time of day, with sleep-induced modulations being particularly evident before sleep onset (00:00–04:00). DST and DPG were significantly, albeit modestly, related to subsequent momentary sleepiness assessments in both sleep conditions, with an increase in DST and a decrease in DPG correlating with increased sleepiness levels. The relationship between skin temperature and subjective fatigue depended on sleep condition, and PST did not correlate significantly with self-reports. Significant effects of sleep restriction were also observed on markers of circadian variation in skin temperature, with an increase in acrophase and a decrease in amplitude of the DST rhythm under the sleep-restricted condition. **Discussion/Conclusions**: Sleep restriction appears to disrupt participants on a thermophysiological level, particularly just before delayed sleep onset. Independently of the sleep condition, the relationship between daytime skin temperature and momentary sleepiness assessment is interesting but merits further investigation. In particular, skin temperature could offer a minimally intrusive method for objectively monitoring or predicting sleepiness levels among individuals with excessive daytime sleepiness diagnoses. The effects of disrupted sleep on circadian rhythm (indexed by skin temperature) were limited by the small number of participants and the duration of data collection. More (and longer) studies are needed to investigate the relationship between sleep restriction, circadian skin temperature rhythm, and the severity of next-day daytime sleepiness.

### 2.47. Impact of Light Illuminance on Locus Coeruleus Activity During an Auditory Emotional Task: Insights from High-Resolution MRI Imaging

Fermin Balda, Roya Sharifpour, Elise Beckers, Islay Campbell, Nasrin Mortazavi, Ekaterina Koshmanova, Puneet Talwar, Laurent Lamalle, Mikhail Zubkov, Gilles Vandewalle, Alexandre Berger, Sara Letot

CRC-Human Imaging, Liege, Belgium

**Abstract:Introduction**: Light not only influences visual functions, but also affects a wide range of physiological processes such as sleep, cognition, and affective state. While the precise neural mechanisms underlying these effects in humans remain unclear, animal studies suggest involvement of subcortical regions, including the locus coeruleus (LC) in the brainstem, which is the main source of noradrenaline in the brain and is implicated in multiple cognitive processes. Here, we investigated how light may affect LC activity during an emotional task using high-resolution MR imaging. **Methods**: A total of 28 young and healthy participants of both sexes (age 24.6 + 3.14y; 17 women) completed a validated auditory emotional task in the morning, 2 h after waking-up time. The task consisted of lure gender classifications of meaningless vocalizations. Untold to the participants, the stimuli were either pronounced with an angry (50%) or neutral (50%) prosody. They were concomitantly exposed to alternating 30 to 40 s blocks of light of different illuminance (0.2, 37, 92, and 190 melanopic EDI), separated by ~10 s intervals in darkness. Meanwhile, functional MRI data were acquired using a 7 Tesla scanner. Separate structural MRI sequences allowed the manual expert delineation of the LC. Individual LC masks were further divided into caudal, medial, and rostral regions and used for extraction of LC responses during the emotional task. **Results**: The statistical analysis consisted of a generalized linear mixed model (GLMM) which revealed that LC activity was associated with significant interactions between light conditions and emotional stimuli (F(3) = 0.298, *p* = 0.017) and between light condition and season (F(3) = −0.362, *p* < 0.001), as well as a main effect of season (F(3) = 1.25, *p* < 0.001). The GLMM did not yield differences between LC subparts. Post hoc contrasts indicated that LC activity increased and decreased under higher illuminance for neutral and emotional stimulations, respectively. These effects were more important during short days compared with long days. Lastly, LC activity was higher under a longer photoperiod. **Conclusions**: The findings show that light modulates LC activity according to the emotional content of incoming stimuli as well as according to seasons. The results illuminate the intricate relationship between ambient light exposure, LC activity, and emotion that may contribute to the longer-term effect of light and light therapy on the affective state.

### 2.48. Dynamic LED Light Versus Static LED Light for Depressed In-Patients: Results from a Randomized Clinical Study

Klaus Martiny ^1^, Carlo Volf ^1^, Anne Sofie Aggestrup ^1^, Paul Michael Petersen ^2^, Carsten Dam-Hansen ^2^, Ulla Knorr ^1^, Ema Erkocevic ^3^, Janus Engstrøm ^3^, Janus Christian Jakobsen ^3^, Helle Østergaard Madsen ^1^, Ida Hageman ^1^

^1^  Mental Health Center Copenhagen, Copenhagen, Denmark
^2^  Technical University of Denmark, Kongens Lyngby, Denmark
^3^  Centre for clinical intervention research, Copenhagen, Denmark

**Abstract:Introduction**: The biological effects of light on human physiology and mental state have been detailed in the last 20–30 years of translational research showing a profound impact on sleep, diurnal rhythms, mood, and alertness. Static electrical lighting is now increasingly being replaced by dynamic lighting in both somatic and mental health hospitals. Dynamic lighting can mimic the naturally occurring seasonal and daily changes in daylight through LED technology. By using light schedules that continuously change in intensity and spectral distribution throughout the 24 h day, we can potentially tailor light to the needs of the human circadian and mood systems. The evidence for a clinical effect of dynamic lighting in mental health hospitals is, however, very sparse, and most studies are of a descriptive non-randomized design. Therefore, there is a need to assess the tolerability and clinical effect of the lighting systems. **Methods**: In the in-patient affective disorders ward, the existing lighting system was replaced in 10 of the 12 bedrooms with a dynamic LED light system that could operate in a dynamic or static mode. Patients were randomly allocated to a static or a dynamic light schedule working in their bedroom. Patients were assessed at baseline and weekly for three weeks. The primary outcome was the change in Hamilton depression rating subscale HAM-D6 scores from baseline to endpoint. Side effects, room occupancy, and sleep were assessed. **Results**: In total, 60 patients were included in the study with a 98.3% follow-up of the primary outcome. The dynamic lighting system had higher satisfaction rates. The primary outcome was negative, but sensitivity analyses with completers with unipolar depression showed a marginal superiority in the Dynamic group on the HAM-D_17_ scale. A better sleep quality (PSQI), longer sleep with fewer awakenings, and a later sleep offset was found in the Dynamic group. No serious adverse events were seen and suicidal scores in the SIDAS scale were low without difference between the groups. Participants were more satisfied with the dynamic lighting than the static and with little glare. **Conclusions**: The dynamic light system was well functioning and well tolerated. A possible antidepressant and a certain sleep improving effect was seen in the Dynamic group. This is a first-time report of sleep improvement from an RCT study using dynamic lighting in a psychiatric in-patient ward. These findings should be tested in larger studies with sleep as the primary outcome.

**Keywords**: major depressive disorder; bipolar disorder; light; sleep; mental health; hospital; antidepressive agents; randomized controlled trial

**Funding:** Danish Energy Foundation, Dr Thorvald Madsen Foundation, Toyota Foundation.

### 2.49. Circadian Variability in Pro- and Antioxidant Defense and Proteolysis in Osteoarthritis, Gout, and Rheumatoid Arthritis: Implications for Sample Timing in Clinical Practice and Research

Oksana Mykytyuk ^1^, Olga Pishak ^2^, Halyna Arych ^3^, Liudmyla Mikulets ^4^

^1^  Bukovinian state medical university, Chernivtsi, Ukraine
^2^  Chernivtsi National University, Chernivtsi, Ukraine
^3^  Mildura Base Hospital, Mildura, Australia
^4^  Bukovinian State Medical University, Chernivtsi, Ukraine

**Abstract:Background**: Rheumatology progress is characterized by numerous discoveries about circadian rhythmicity in healthy state and pathology over the last decades. Despite this, routine testing in everyday clinical practice, and even with research samples, is still performed at the traditional time, mostly morning hours. We performed an evaluation of the daily variability of some parameters characterizing pro- and antioxidant defense and proteolysis in patients with osteoarthritis, gout, and rheumatoid arthritis vs. a healthy population in order to define whether a classic sample time is the most representative of possible pathological changes. **Methods**: A total of 88 patients with osteoarthritis, 60 patients with gout, and 60 with rheumatoid arthritis vs. 40 healthy people were investigated after obtaining informed consent on their first day in clinic after hospitalization due to exacerbation of their joint pathology. A full clinical examination with an estimation of the daily variability of symptoms was performed. Levels of malonic dialdehyde, oxidatively modified proteins’ products, glutathione restored, catalase activity, and collagenolytic activity of blood plasma were checked applying standard biochemical methods at 4 h intervals (starting from 10.00 a.m.). All the results were checked statistically. **Obtained Results**: Healthy people demonstrated clear circadian variability of estimated indices. They had higher levels of prooxidant values and lower ones of antioxidant defense factors during the light period of the day, coinciding with physical activity. In those with osteoarthritis, we observed a shift in peak values of malonic dialdehyde, collagenolytic activity of blood plasma, and the lowest levels of antioxidant defense indices at evening and early night hours (10.00 p.m–2.00 a.m.), in parallel with the most intensive pain. Morning values were the same as in the healthy individuals. Patients with gout demonstrated a significant increase in prooxidant values at morning and daytime hours (10.00 am–6.00 p.m.). Antioxidant defense was lowest at the same time. Rheumatoid arthritis patients were characterized by the shift in timing of the highest activity of prooxidant processes and collagenolysis in early morning hours (2.00–6.00 a.m.) with the lowest activity of the antioxidant defense systems. **Conclusions**: This study demonstrates significant circadian variability in pro- and antioxidant defense parameters and proteolysis among patients with osteoarthritis, gout, and rheumatoid arthritis compared with healthy individuals. Notably, the timing of peak values varied with each condition. These findings suggest that the traditional morning sampling time may not be the most representative for detecting pathological changes in osteoarthritis and rheumatoid arthritis. Consequently, adjusting sample collection times to align with the circadian rhythms of specific pathologies could enhance the accuracy and relevance of clinical and research assessments.

### 2.50. Natural Daylight During Office Hours Improves 24 h Glucose Control and Substrate Metabolism in Type 2 Diabetes Patients

Jan-Frieder Harmsen ^1^, Ivo Habets ^2^, Marit Kotte ^2^, Merel Timmermans ^2^, Dzhansel Hashim ^2^, Soraya de Kam ^2^, Gert Schaart ^2^, Anne Gemmink ^2^, Marijke Gordijn ^3^, Patrick Schrauwen ^2^, Charna Dibner ^4^, Joris Hoeks ^2^

^1^  RWTH Aachen University, Aachen, Germany
^2^  Maastricht University, Maastricht, The Netherlands
^3^  University of Groningen, Groningen, The Netherlands
^4^  University of Geneva, Geneva, Switzerland

**Abstract:Background**: Of our time, 90% is spent indoors, where we are exposed to artificial light with lower light intensities and a different color spectrum compared with natural daylight. This chronic lack of natural daylight is increasingly being considered a risk factor for metabolic diseases, such as type 2 diabetes (T2D). Here, we investigated the potential benefit of increasing the amount of time spent in natural daylight exposure compared with artificial lighting on 24 h glucose control and substrate metabolism in T2D patients. **Methods**: Thirteen T2D patients (mean ± SD, 70 ± 6 years, BMI: 30.1 ± 2.3 kg/m^2^) were exposed to two lighting interventions of 4.5 days in a randomized crossover design. In one study arm, natural daylight was facilitated through windows, whereas in the other arm, participants were exposed to constant artificial lighting (photopic illuminance: 300 lux, melanopic EDI: 209 lux) during office hours (8:00–17:00 h). A washout period of at least 4 weeks separated both conditions. Evenings were spent in dim light (<5 lux) and the sleeping period occurred in darkness (23:00–7:00 h) in both arms. Volunteers were provided with standardized meals and wore continuous glucose monitors (Abbott, Freestyle Libre Pro iQ) on their upper arms. On day 4, indirect calorimetry was performed every 5 h around the clock to assess 24 h substrate metabolism and energy expenditure. In parallel, frequent blood draws were conducted to assess circulating metabolites. On the evening of day 4, saliva samples were collected every 30 min from 19:00 to 23:00 h to determine dim light melatonin onset (DLMO). Core body temperature was measured using a telemetric pill. On day 5, a fasted muscle biopsy was taken to assess clock gene expression, and a mixed meal test (MMT) was executed, for which frequent blood samples were taken in conjunction with indirect calorimetry. **Results**: The time spent in the normal glucose range (4.4–7.2 mmol/L) was higher upon natural daylight compared with artificial light (50.9 ± 21.5% vs. 43.3 ± 23.8%, *p* = 0.036). The respiratory exchange ratio (RER) during daytime was lower in the natural light condition, indicating a shift towards fat metabolism in the natural compared with the artificial light condition (*p* = 0.029). In accordance, the RER during the MMT was also lower upon natural daylight (*p* = 0.04). Salivary melatonin levels were higher in the late evening (21:00–23:00 h) upon natural daylight, but DLMO was not different between conditions (natural: 20:44 ± 01:26 h vs. artificial: 21:11 ± 02:11 h, *p* = 0.338). The mRNA levels of *Per1* (*p* = 0.01) and *Cry1* (*p* = 0.021) in skeletal muscle were higher upon natural daylight. The diurnal rhythm in core body temperature was not different between light conditions. **Conclusions**: Our findings suggest that natural daylight exposure during office hours has a positive impact on metabolism and could support the treatment and prevention of metabolic diseases.

### 2.51. A Case Series of a Non-24-Hour Sleep–Wake Disorder in the Sighted (s-N24SWD) Description of a Psychiatric Phenotype

Karin van Rijn ^1^, Marijke Gordijn ^2^

^1^  SEIN, The Netherlands
^2^  University of Groningen, Groningen, The Netherlands

**Abstract:** A non-24 h sleep–wake disorder (N24SWD) in the sighted is presumed to be rare (Garbazza, 2018). In this series of clinical case studies, the preliminary results of diagnostics and treatment of s-N24SWD are discussed. It shows that s-N24SWD is highly related with neurobiological disorders, and young males with an autism spectrum disorder (ASD) especially seem to be at risk. **Methods**: From 2018 to now, 76 adults (≥18 years) were diagnosed with an s-N24SWD. Diagnostics were either 2–3 weeks of actigraphy alone or actigraphy in combination with a repeated dim light melatonin onset (DLMO) assessment. Diagnostics for other sleep disorders like restless legs (RLS) and sleep apnea (OSAS) were assessed with polysomnography (PSG). Additional medical and psychiatric history was obtained as well as daily activities, vitamin D levels, meal timing and light-dark exposure. Prior to entrainment therapy, vitamin D levels were tested and in case of deficiency, supplemented patients were treated according to a protocol in order to implement Zeitgebers like light and dark, meal timing, and exercise. A chronotherapeutic dosage of melatonin (0.5 mg) was started 5 h before bedtime when the patient was close to the desired time frame for entrainment. **Results**: Three patients were excluded for further analysis due to another etiology, e.g., bi-polar disorder and pituitary problems. The majority of patients (n = 73) with s-N24SWD were male (63 m vs. 10 f). The average age was 26.6 years (range 18–57y), and 99% had a prior delayed sleep–wake phase disorder. Most patients (n = 62) were diagnosed with a neurodivergent disorder (ADHD and/or ASS). Thirty patients (41%) were able to maintain an entrained sleep–wake rhythm after treatment up to a year after treatment. In total, 12 patients (16%) stopped during or did not start treatment. For 31 patients (42%), treatment is ongoing. Data prior to and after treatment from six patients will be presented. The case studies show a different development of s-N24SWD versus N24SWD in the blind. In the blind, there was a slow, gradual shift in the sleep period, mostly a delay. In the sighted the shift could be gradually delayed, but quite often there were larger phase jumps forward. These were followed either by a shift backwards or a relative coordination period. The vast majority of the patients had a vitamin D deficiency and a disrupted pattern regarding meal timing. **Conclusions**: Although there are similarities between a N24SWD and s-N24SWD, s-N24SWD can be interpreted as a subclass of the intrinsic circadian rhythms sleep–wake disorders, due to a different etiology than in the blind, the high amount of psychiatric co-morbidity, and the role of a long endogenous period. Contributing factors to s-N24SWD seem to be low vitamin D levels, excessive gaming and poor meal timing. In order to achieve an entrained sleep–wake rhythm, close collaboration with other professionals like psychiatrists, dieticians, and case workers is needed.

### 2.52. Behavior-Based Movement Cut-Off Points in 3-Year-Old Children Comparing Wrist and Hip-Worn Actigraphs MotionWatch 8 and ActiGraph GT3X

Katharina Wulff, Daniel Jansson, Rikard Westlander, Magnus Domellöf

Umeå University, Umeå, Sweden

**Abstract:Background**: Movement-related assessments of habitual activities, such as level of physical activity (PA) or timing of sleep/circadian rhythms, have typically taken a segregated rather than integrated approach across research disciplines such as sports medicine and chronobiology. The evidence base is similarly segregated, and evidence concerning combining movement behaviors that constitute the entire 24 h period using compositional analyses is, to date, rather uncommon. To apply entire 24 h analyses combining sleep and PA, we aimed at (i) calibrating activity counts of motor behavior measured simultaneously with MotionWatch 8 (MW8) and ActiGraph (GT3X) in 3-year-old children, (ii) documenting movement intensities in 30 s epochs at wrist/hip positions, and (iii) we evaluated the accuracy of cut-off agreements between different behavioral activities. **Methods**: Thirty 3-year-old children of the NorthPop cohort performed six directed behavioral activities individually, each for 8–10 min while wearing two pairs of devices at hip and wrist positions. Directly observed naturally occurring behaviors included watching cartoons, recumbent story listening, sitting and handcrafting, floor play with toys, engaging in a brisk walk, and a sprinting game. Receiver operating curve classification was applied to determine activity thresholds and to assign activity composite classes from context-guided behaviors. **Results**: Activity counts of MW8 and GT3X pairs of wrist-worn (r = 0.94) and hip-worn (r = 0.79) devices correlated significantly (*p* < 0.001). Activity counts at hip position were significantly lower compared with those at the wrist position (*p* < 0.001), irrespective of device type. Sprinting, floorball/walk, and floor play assigned as “physically *mobile*” classes achieved outstanding accuracy (AUC > 0.9) and two sedentary and a motionless activities assigned into “physically *stationary*” classes achieved excellent accuracy (AUC > 0.8). **Conclusions**: This calibration provides useful cut-off thresholds for physical activity levels of preschool children. The contextual information and proportions of physical activity can now be scaled and integrated into projects on sleep, circadian rhythms, and light exposure.

**Keywords**: accelerometer; actigraphy; motor development; preschool

### 2.53. Sex and Seasonal Changes in Human Melatonin Suppression and Alerting Response to Light

Fatemeh Fazlali ^1^, Rafael Lazar ^1^, Oliver Stefani ^2^, Manuel Spitschan ^3^, Christian Cajochen ^1^

^1^  University of Basel, Research Cluster Molecular and Cognitive Neurosciences, Basel, Switzerland
^2^  Lucerne University of Applied Sciences and Arts, School of Engineering and Architecture, Horw, Switzerland
^3^  Technical University of Munich, Translational Sensory & Circadian Neuroscience, Munich, Germany

**Abstract:Background**: Evidence suggests that sensitivity to non-image forming (NIF) effects of light varies by individual differences such as sex and seasonal changes. It is unclear, however, whether this applies to the levels of light we normally experience in front of visual displays in the evening. There is evidence that females are more sensitive to bright light, which affects melatonin secretion and circadian rhythms more than males. Seasonality also has an impact on light sensitivity and circadian regulation, with reduced sensitivity to light and an earlier circadian phase in the summer compared with the winter in mid-latitude regions. Thus, here we examined if the melatonin suppression and alerting response to moderate light levels is modulated by sex, seasons, and their interaction. **Methods**: In a within-subjects design, 48 healthy young adults (aged 18–35 years, 50% female) were recruited across different seasons in a year. The study design included two 9 h laboratory sessions, with at least 5 days of washout between sessions. Each session began 6 h before the participant’s bedtime and ended 3 h after. Prior light history and sleep–wake rhythms were monitored using wrist actimetry. Menstrual phase was reported using a self-reported questionnaire. Participants were exposed to dim light (~8 lx) and moderate light (~100 lx) for 2 h after their habitual bedtime. Salivary melatonin samples and subjective sleepiness ratings were collected at 30 min and hourly intervals, respectively. The effects of prior light history (i.e., duration of the time above the threshold (TAT) 100 lx) and menstrual phase were also assessed in the analysis. Statistical analyses were performed using linear mixed models to examine the effects of sex, season, and their interaction on sensitivity to moderate light compared with dim light. **Results**: Female participants showed greater melatonin suppression but a lower alerting response to moderate light compared with males. Sensitivity to NIF of moderate light was greater in winter than in summer, as evidenced by stronger melatonin suppression and higher alertness in both sexes compared with summer. Notably, the interaction of light exposure, season, and sex tended to be significant in terms of melatonin suppression. Prior light history had no significant effect on melatonin suppression and alertness, but did predict the dim light melatonin onset (DLMO) in both summer and winter. **Conclusions**: The results indicated that female participants were more sensitive to light-induced melatonin suppression than male participants, although this was not the case for light-induced alertness changes. Both sexes showed stronger NIF effects in winter compared with summer. Furthermore, a longer prior light exposure above 100 lx was associated with earlier DLMO. These findings highlight the importance of considering sex, seasonality, and the combination of both in light sensitivity and emphasize the necessity of customizing light exposure recommendations to improve sleep quality and manage circadian rhythm disorders.

**Keyword**: light sensitivity; subjective sleepiness; prior light history; dim light melatonin onset; non-image forming effects

**Funding:** This study is part of the European Training Network, supported by the European Union’s Horizon 2020 Program under the Marie Skłodowska-Curie grant agreement No. 860613 (LIGHTCAP).

### 2.54. Interstimulus Interval and Its Impact on the Pupillary Light Reflex

Simon Belgers, Raymond Cuijpers, Wenger Pwa, Ingrid Heynderickx

Human Technology Interaction group, department of Industrial Engineering and Innovation Sciences, Eindhoven University of Technology, North Brabant, The Netherlands

**Abstract:Background**: The Pupillary Light Reflex (PLR) serves as an important tool for both research and diagnostic purposes. The PLR shows pronounced differences between light pulses of high biological potency and those of low biological potency. Although further research is warranted, it is clear that the pupillary light response is influenced by prior light exposure. Some research has been carried out on the approximate duration of a steady state pupil size after a brief light pulse. Ideally, methodologies should include at least sufficient time for the pupil to reach a steady state between each individual measurement. This requirement, however, contrasts with the need to limit the duration of each session to reduce participant burden and reduce confounds. To collect more data per session, it may be beneficial to start a subsequent measurement before the pupil reaches a steady state. The impact of this shortened interstimulus interval on the effect of biological potency on the light response is currently unclear, as is its potential benefit. Our experiment involved seventeen participants, during which we recorded four sequences of fifteen pupil measurements and extracted the Post-Illumination Pupil Response (PIPR), a commonly used measure of the PLR. Two sequences allowed a sufficient interstimulus interval for the pupil to reach a steady state, while the other two sequences involved more rapid interstimulus intervals. Both sets of sequences were conducted with metameric light, with each second sequence exhibiting increased biological potency. Our findings indicate that the time between measurements reduced the PIPR. Additionally, the PIPR increased at later measurements within a sequence, but only under conditions with a short time between measurements. We found no evidence of an effect of biological potency on the PIPR. These results suggest that the interstimulus interval impacts the PLR. Given that only two unique interstimulus intervals were studied, it remains unclear whether there is a minimum interstimulus interval beyond which an increased interval no longer affects the PLR. We also found that the measurement number was significant only when using a short interstimulus interval, and it is of interest at what interstimulus interval this effect of subsequent measurements on the PLR is no longer of significance. Contrary to the existing literature, we found no effect of biological potency, likely due to an insufficient contrast between conditions.

**Keywords**: post-illumination pupil response; pupillary light reflex; metameric light; melanopic equivalent daylight illuminance; interstimulus interval

**Funding:** This work was (partially) supported by funds from the European Union and the nationals contributing to the context of the ECSEL Joint Undertaking program (2021–2024) under the grant #101007319.

### 2.55. Circadian Disruption and Persistent Sleep Disorders in Osteoarthritis: Implications for Comprehensive Management

Oksana Mykytyuk

Bukovinian State Medical University, Chernivtsi, Ukraine

**Abstract:Background**: Osteoarthritis is a disorder of interest in the circadian medicine field. It was proven in numerous studies that this disease is characterized by symptoms with a marked daily variation: maximal intensity of pain at evening and night hours. That is a reason for subsequent sleeping disorders and possible social jet lag. Pharmacological treatment of osteoarthritis is a complicated task for rheumatologists. Strategies includes using painkillers and anti-inflammatory drugs for a quick relief, topical therapy, or chondroprotectors. Minimal attention is given to the estimation of circadian misbalance and its correction in a mentioned population. **Methods**: In total, 120 patients with primary osteoarthritis of lower extremities joints (knees, hips, ankles) (female–male ratio 3:1, average age 57.3 ± 9.2, 2nd–3rd grade by Kellgren–Lawrence) were investigated after obtaining informed consent on their first days in clinic after hospitalization due to exacerbation of their joint pathology, and before discharge. A full clinical examination with an estimation of the daily variability of symptoms was performed. Patients were asked to fill in the WOMAC scale for Osteoarthritis estimation, visual analog pain scale (VAS) 6 times per day, sleep diaries, and the Epworth Sleepiness Scale (ESS). Assessment of Insomnia and Sleep was carried out using the Insomnia Severity Index. **Obtained Results**: A total of 81.4% patients suffered from pain of moderate-to-severe intensity during the daytime (average VAS score—6.4 ± 1.3), but marked significant pain increase predominantly during evening and night hours (average VAS score—8.1 ± 0.8), especially after physical loading. Of the investigated patients, 92.3% reported chronic insomnia due to pain. They marked a necessity to wake up, switch on the light to find rescue medication, make efforts to distract themselves watching late-night TV shows, etc. That was confirmed by data from the questionnaires. AT the end of the 10-day course of treatment that included NSAIDS, physiotherapy, and symptomatic treatment, 78.7% patients reported significant improvement during the daytime, and 63.7% patients mentioned the relief in the evening time. Despite that, almost all patients who had sleep disorders on admission reported difficulties of falling asleep and frequent awakening, not related to pain, even after treatment. **Conclusions**: This study highlights the significant impact of circadian disruption on the symptomatology and management of osteoarthritis. Despite effective daytime symptom management with NSAIDs, physiotherapy, and other symptomatic treatments, evening and nighttime pain remain a significant issue, leading to chronic insomnia and potentially contributing to social jet lag in this population. The findings suggest that current osteoarthritis management strategies may be insufficient without considering the circadian aspect of the disorder. Incorporating circadian medicine principles, such as tailored timing of medication and therapeutic interventions, could enhance overall treatment effectiveness and improve quality of life for patients.

### 2.56. Effects of Light from Electronic Devices on Self-Reported Sleep in the U.S. Population

Shadab Rahman ^1^, Salim Qadri ^2^, Michael Herf ^3^, Lorna Herf ^3^, Leilah Grant ^1^, Melissa St. Hilaire ^1^, Steven Lockley ^1^

^1^  Harvard Medical School, Boston, MA, USA
^2^  Brigham and Women’s Hospital, Boston, MA, USA
^3^  F.lux Software LLC, Los Angeles, CA, USA

**Abstract:Background**: Sleep deficiency is common in modern society and increases the risk of accidents, injuries, and poor health. Up to 90% of people use electronic light-emitting devices (ELEDs) in the evening up to 1 h before bedtime. Evening light exposure directly alerts the brain and delays the circadian timing of sleep. Since these photic responses are preferentially sensitive to short-wavelength light, engineering the spectral characteristics and illuminance of ELED screens may attenuate the disruptive effects of evening light exposure on sleep. **Methods**: We examined the impact of reducing the short-wavelength content and illuminance of personal computer screens between local sunset and bedtime using the F.lux^®^ software on self-reported sleep duration, efficiency, and bedtime from daily logs. Participants were recruited across the U.S. and all data collection was performed online. Adults ≥18 years of age who downloaded the software were asked to participate. In total, 15,526 participants consented to participate and were randomized to 1 of 6 screens correlated color temperature (CCT) conditions (1900 K [least short-wavelength content], 2700 K, 3400 K, 4100 K, 4800 K, 5600 K [most short-wavelength content], a relative reduction of 90–15% in melanopic irradiance, respectively). Amongst those who consented, a subset of participants (12,565) provided objectively assessed data on device-usage timing. Of those who consented, 5323 participants provided longitudinal data on sleep, on average (±SD), for 5.8 ± 4.2 days (total 35,323 nights) with complete data on predefined covariates outlined below. Group differences in self-reported sleep duration, sleep efficiency, and bedtime by CCT condition were analyzed using generalized linear mixed models with subject-level random effects, adjusting for objectively assessed device usage in the last five hours preceding bedtime, age, sex, race, device platform, sleep disorders diagnosis, and self-reported shift work. **Results**: At baseline, self-reported average (±SD) sleep duration was 7.2 ± 1.4 h, sleep efficiency was 92.4 ± 8.3%, bedtime was 23:48 ± 2:00 h, and device usage averaged 1.2 ± 1.3 h in the 5 h preceding sleep. Sleep disorders were reported in 34.8% (n = 1854) and shift work in 20.0% (n = 1066) of participants. In daily device-usage timing, the circular mean of activity was 26 min earlier in the 1900 K compared with the 5600 K condition (*p* < 0.0001). Although there was no direct independent effect of CCT on sleep duration (*p* = 0.10), efficiency (*p* = 0.80), or bedtime (*p* = 0.56), there was a significant effect modification based on device usage in the last five hours prior to sleep. Overall, sleep duration was significantly shorter (interaction *p* = 0.02), efficiency was significantly lower (interaction *p* = 0.04), and bedtime trended to be later (interaction *p* = 0.07), with more device usage in the last 5 h before bedtime in the 5600 K condition but not in the 1900 K condition. Sleep duration, efficiency, and bedtime all varied significantly based on sex, age, and race (all *p* < 0.01). Moreover, the interaction effect between condition and device usage on sleep duration and efficiency, but not bedtime, was further modified by sex and age (both four-way interaction *p* < 0.03). **Conclusions**: Our results show that electronic device usage prior to bedtime disrupts sleep in the general population, which may be attenuated by reducing the illuminance and short-wavelength content of light emitted from these devices. Age, sex, and race are important effect modifiers, which may impact interindividual differences in response to light exposure and need to be considered for optimizing photic interventions.

### 2.57. Exposure to Bright Light During Afternoon to Early Evening Reduces Later Evening Melatonin Release in Adolescents

Rafael Lazar ^1^, Fatemeh Fazlali ^2^, Oliver Stefani ^3^, Manuel Spitschan ^4^, Christian Cajochen ^5^

^1^  University of Basel, Research Cluster Molecular and Cognitive Neurosciences, Basel, Switzerland
^2^  Psychiatric University Clinics (UPK) Basel, Centre for Chronobiology, Basel, Switzerland,
^3^  Lucerne University of Applied Sciences and Arts, Luzern, Switzerland
^4^  Technical University of Munich, Munich, Germany
^5^  University of Basel, Research Cluster Molecular and Cognitive Neurosciences, Basel, Switzerland

**Abstract:Background**: During the school year, adolescents are particularly susceptible to sleep deprivation due to a combination of early school start times and late bedtimes, often related to a delay in circadian timing during puberty. Additionally, late evening light exposure can contribute to later bedtimes by acutely reducing sleepiness and melatonin release, and further delaying circadian timing. Evidence from several studies suggests that an individual’s light exposure history may moderate physiological responses to subsequent light at night. However, it remains unknown how this adaptation depends on the intensity and timeframe of the prior light history. The objective of this study was to investigate whether increasing light exposure after school hours could be a viable approach to reducing the alerting effects of artificial light near bedtime. In a counterbalanced crossover study, we tested how recent afternoon to evening light history influences melatonin levels, sleepiness, and vigilance test performance during later evening light exposure in adolescents. **Methods**: Twenty-two healthy adolescents (14–17 years) participated in a 3-week study schedule consisting of 3 in-lab experimental sessions starting 8 h before their habitual bedtime (HBT; 18.5 h protocol), preceded by 5 days of ambulatory sleep–wake and light exposure monitoring. Laboratory measurements included salivary melatonin (AUC), subjective sleepiness (KSS), and vigilance test performance (PVT). All experimental sessions consisted of the same late evening light exposure starting 3 h before HBT (duration: 4.5 h, ~80 lx mEDI, 4000 K), preceded by 1 of 3 4 h light intervention conditions (4000 K, different intensities) starting 7.5 h before HBT: (A) dim ~4 lx; (B) moderate ~80 lx; or (C) bright ~1400 lx mEDI. We performed linear mixed model analyses (α = 0.05) to estimate the effects of moderate and bright compared with the dim afternoon light intervention. Self-reported chronotype, pubertal stage, and wrist-recorded light history (time above threshold, TAT > 1000 lx) from a day before the experiment were modeled as fixed effect covariates, and repeated measurements per participant as a random intercept. **Results**: During the bright and moderate afternoon light intervention subjective sleepiness was significantly reduced compared with dim light. Contrary to our hypothesis, melatonin levels were significantly lower when the evening light exposure was preceded by the bright compared with the dim light intervention, whereas subsequent evening sleepiness and vigilant attention were not significantly affected. Contrasting these results, increased bright light exposure from a day before the in-lab session was significantly associated with increased evening melatonin levels and sleepiness. **Conclusions**: Our data suggest that increasing the very recent bright light history of adolescents up to 3 h before HBT may be inappropriate for promoting higher melatonin release in moderate light on the same evening. Conversely, increased exposure to bright light from the preceding day (TAT > 1000 lx) may effectively reduce the alerting effects of light and increase melatonin levels the following day(s). This may imply that the light-history-driven adaptation of circadian photosensitivity takes longer than we initially hypothesized.

**Keywords**: Melatonin; adolescents; light history; sleepiness; crossover design

**Funding:** EU-ITN LIGHTCAP project (860613—H2020-MSCA-ITN-2019).

### 2.58. Effectiveness of Individualized Chronotherapy in Individuals with Subclinical Sleep Problems

Katarína Evansová ^1^, Kateřina Červená ^2^, Ondřej Vaníček ^3^, Marijke Gordijn ^4^, Jana Kopřivová ^5^

^1^  Third Faculty of Medicine, Charles University, Prague, Czech Republic
^2^  Department of Molecular Biology, Umeå University, Umeå, Czech Republic
^3^  Czech Republic-Department of Psychology, Faculty of Arts, Charles University, Prague, Czech Republic; Sleep and Chronobiology Research Centre, National Institute of Mental Health, Klecany, Czech Republic
^4^  University of Groningen, Groningen, The Netherlands
^5^  National Institute of Mental Health, Prague, Czech Republic

**Abstract:Background**: The current situation has a negative impact on the sleep and psychological well-being of a large part of the population, which includes people with undiagnosed but limiting sleep problems. These sleep difficulties are often subsequently addressed with drugs that can quickly become addictive and very often do not address the cause but only the symptoms. In this research, we therefore focused on a non-pharmacological chronotherapeutic intervention that combines bright light phototherapy, the use of glasses that filter the blue component of the light spectrum, education about proper sleep hygiene, and individual adjustment of the daily routine in relation to wake-up time, daily activities, and timing of sleep according to the individual’s chronotype and preferences. Different forms of this intervention were examined in three groups to determine effect sizes. **Methods**: The effect of chronotherapy was measured in 3 groups: (1) Experimental group, which included phototherapy, blue-light blocking glasses, individual sleep schedule according to chronotype, and sleep and light education. (2) Semi-placebo group, which received glasses with clear lenses (no filter), individual sleep schedule according to chronotype, and sleep and light education. (3) A control group that received no aids or education, only completed questionnaires. All groups simultaneously completed questionnaires focusing on sleep (PSQI, ISI, ESS, MEQ), mood (BDI-II and BAI), and completed sleep diaries. They completed the questionnaires before entering the study, after chronotherapy and 4 weeks after the end of chronotherapy to determine the duration of the effect. Completion of the questionnaires reflected the timing of the experimental and semi-placebo groups, i.e., at the beginning, during, and at the end of this study. **Results**: A sample of 52 subjects has been obtained, 20 in the experimental group, 19 in the semi-placebo group, and 13 in the control group. Significant effects were found for sleep questionnaires (PSQI, ISI), mood (BDI-II, BAI), and also for the sleep diaries (Latency, Efficiency). CONCLUSIONS: The data show significant changes in both the experimental and semi-placebo groups compared with the control group. Interestingly, sleep and mood improved in both the experimental and the semi-placebo groups, almost equally, despite the fact that the semi-placebo group did not receive phototherapy and the glasses were filter-free, i.e., placebo. These results show that even interventions without aids such as individual sleep schedules that respect the individual’s chronotype, together with sleep and light education, have a significant effect on both sleep and mood in individuals with subclinical sleep problems.

1. **Acknowledgements:** Charles University Grant Agency grant no. 355322. (Effectiveness of individualized chronotherapy in individuals with subclinical sleep problems).

### 2.59. Personalized Light Therapy for Night Shift Workers: A Precision Medicine Approach to Reducing Insomnia and Sleepiness

Philip Cheng ^1^, Olivia Walch ^2^, Marleigh Treger ^1^, Jonny Russell ^1^, Christopher Drake ^1^

^1^  Henry Ford Health + Michigan State University Health Sciences, USA
^2^  Arcascope, Arlington, VA, USA

**Abstract:Introduction**: Night shift workers experience symptoms of excessive sleepiness and insomnia due to misalignment between their circadian clock and work schedule. Circadian misalignment can be corrected using exposure to bright light delivered in accordance with a phase response curve. Our prior data indicate that a light schedule personalized to an individual’s melatonin rhythms produces greater reductions in circadian misalignment compared to a one-schedule-fits-all approach. This randomized controlled trial extends prior findings by examining the effect of personalized light therapy on symptoms of shift work disorder. **Methods**: Individuals with shift work disorders (ICSD-3 diagnostic criteria) were randomized into two conditions: personalized light therapy (n = 14) or non-personalized light therapy control (n = 7). Personalized light schedules were based on estimates of dim light melatonin onset (DLMO) derived from mathematical modeling of data collected via an Apple Watch. Light schedules were delivered through a mobile app (Arcashift) that updated in accordance with real-time estimates of DLMO. Estimates were confirmed with in-lab DLMO. Participants were provided light blocking glasses and a light box as source of bright light at night. Sleepiness (Karolinska Sleepiness Scale) and insomnia (Insomnia Severity Index) were assessed before and after treatment, and analyses evaluated change scores from pre- to post-treatment. **Results**: Those in the personalized light therapy group demonstrated decreased insomnia symptoms during daytime sleep (mean = −4.64, SD = 8.03) compared with those in the non-personalized control (mean = 3.57, SD = 5.38), *p* < 0.05. The personalized light therapy group also achieved a decrease in peak sleepiness (mean = −0.21, SD = 0.68) compared with the control (mean = 0.77, SD = 0.76), *p* < 0.001. **Conclusions**: Preliminary results suggest that personalizing light therapy according to the individual’s specific circadian phase may be more effective in improving symptoms of insomnia and sleepiness by delivering treatment. Future research should examine other occupational and health outcomes associated with a personalized approach to light therapy.

### 2.60. Timed Exercise as a Tool to Improve Aberrant Circadian Function

Alun Hughes ^1^, Timna Hitrec ^2^, Cheryl Petit ^3^, Beatriz Bano Otalora ^3^, Hugh Piggins ^4^

^1^  Liverpool John Moores University, Liverpool, UK
^2^  University of Bologna, Bologna, Italy
^3^  University of Manchester, Manchester, UK
^4^  University of Bristol, Bristol, UK

**Abstract:** Aberrant circadian rhythmicity has been associated with a wide variety of health concerns, including cardiovascular and metabolic disorders, impaired cognitive function, and poor mental health. As such, identifying mechanisms to stabilize and improve inappropriate circadian function is an important avenue for research aiming to improve human health. Animal models can provide fundamental insights into the mechanisms of human disease and help to identify potential new therapeutic interventions. In the suprachiasmatic nuclei (SCN) of the hypothalamus, the dominant central circadian pacemaker in mammals, neuropeptide signaling via vasoactive intestinal polypeptide (VIP), and its cognate receptor VPAC_2_ is crucial for maintaining appropriate circadian function. Mice lacking this key SCN neuropeptide-signaling system (*Vipr2^−/−^* mice) exhibit altered rhythms in behavior, SCN electrical activity, and SCN clock gene expression, making them a valuable model for the study of circadian dysfunction. Though *Vipr2^−/−^* mice respond poorly to photic entrainment cues, timed daily exercise in a home-cage running wheel promotes persistent near 24 h behavioral rhythmicity in this model, and we recently reported that this is associated with improved SCN rhythmicity and intercellular synchrony. We hypothesized that these improvements may be mediated by timed exercise-induced changes in GABAergic signaling, the main classical neurotransmitter system in the SCN, and increased expression of core circadian clock genes. Using extracellular electrophysiology, RNA-Seq and RTqPCR, we found that though GABA signaling was markedly altered in *Vipr2^−/−^* SCN, it was only modestly impacted by timed exercise in these mice. Moreover, whilst gross expression of core circadian clock genes was reduced in the *Vipr2^−/−^* SCN, this was also not overtly improved by timed exercise. Intriguingly, expression of genes involved in intracellular signaling pathways known to be important for circadian rhythmicity (mTOR and MAPK pathways), as well as genes involved in epigenetic processes, was upregulated. This suggests that the improvements in behavioral rhythms and SCN functionality induced by timed exercise may be sustained by subtle changes in cellular functionality in the SCN, rather than overt changes in the expression of the core circadian molecular machinery or classical SCN neurochemistry. These findings highlight the potential of timed exercise as a novel tool to improve circadian rhythms, identify further avenues for research into the mechanisms of timed exercise-induced circadian improvement, and illustrate the utility of animal models for the identification of potential new treatment strategies for circadian dysfunction.

### 2.61. Effects of a Brief Chronotherapeutic Intervention in Emerging Adults with Delayed Sleep Timing and Depression: Preliminary Findings

Delainey Wescott, Kathryn Roecklein

University of Pittsburgh, Pittsburgh, PA, USA

**Abstract:Background**: Chronotherapeutic interventions can improve depression, suggesting advances in sleep and circadian timing are potential antidepressant mechanisms. However, the field lacks true tests of mechanistic mediation for chronotherapeutic interventions, including pre-, mid-, and post-treatment measures. The current pilot study tested the effectiveness of a chronotherapeutic intervention for individuals with delayed sleep timing and depression symptoms and evaluated whether advances in sleep and circadian timing are underlying mood-improving mechanisms. **Methods**: Emerging adults (ages 18–25) with late sleep times (>1 AM) and mild-to-moderate current depression underwent a 2-week chronotherapeutic manipulation. Sleep times were confirmed with actigraphy, and depression symptoms were determined using a semi-structured interview (SCID-5). Participants wore Re-Timer bright light glasses for 30–60 min upon waking, blue light-blocking glasses for 2 h prior to bedtime and underwent a sleep advancing protocol. Circadian phase was measured pre- and post-manipulation on Friday evenings using the dim light melatonin onset (DLMO). Participants completed questionnaires on circadian preference (Composite Scale of Morningness, CSM; Morningness Eveningness Questionnaire, MEQ) and depression symptoms (Quick Inventory of Depression Symptomatology; QIDS) pre-, mid-, and post-manipulation and completed daily online sleep diaries. We used multilevel models with a participant-level intercept to test the main effect of time on treatment outcomes. We used repeated measures correlation with *rmcorr* in R to measure the correlations between circadian preference and depression symptoms throughout the manipulation. **Results**: Fifteen participants have completed the manipulation to date. Over the two-week manipulation, circadian phase advanced by ~47 min (b = −0.79, *p* = 0.01), sleep diary sleep onset advanced by ~75 min (b = −1.3, *p* < 0.001), and wake time advanced by ~51 min (b = −0.87, *p* < 0.001). Circadian preference shifted towards morningness (CSM: b = 4.6, *p* < 0.001; MEQ: b = 7.6, *p* < 0.001), and depression symptoms decreased (b = −5.2, *p* < 0.001). Sleep duration increased by ~25 min (b = 0.41, *p* = 0.007), frequency of naps decreased (b = −0.28, *p* = 0.003), and self-reported sleepiness decreased (b = −2.3, *p* = 0.01). Notably, improved depression was associated with shifts towards morningness (r_rm_ = −0.52 to −0.62), earlier sleep onset (r_rm_ = 0.45 to 0.63), earlier wake times (r_rm_ = 0.54 to 0.63), and earlier circadian phase (r_rm_ = 0.32 to 0.57). **Conclusions**: Preliminary results suggest the chronotherapeutic intervention successfully advanced sleep and circadian timing and reduced depressive symptoms. Shifts towards morningness were associated with improved depression, which may reflect advanced circadian preference and/or morning alertness as antidepressant mechanisms. These preliminary findings are limited by a small sample size and the lack of a control group. Employing true mechanistic tests are critical for understanding the antidepressant effects of chronotherapeutic interventions.

### 2.62. Optimizing Daytime Light Exposure for Sleep–Wake Consolidation: Insights from Actigraphy

Renske Lok, Jamie Zeitzer

Stanford University, Stanford, CA, USA

**Abstract:Introduction.** Consolidated sleep is crucial for maintaining both physical health and mental well-being. However, as individuals age, their ability to sustain consolidated sleep periods declines, leading to fragmented sleep–wake patterns and increased daytime sleepiness. The underlying mechanisms behind these age-related sleep changes are not fully understood, but it has been postulated that the degeneration of the circadian clock in the hypothalamic suprachiasmatic nuclei may play a pivotal role in disrupting sleep–wake patterns. One explored countermeasure to sleep–wake fragmentation is increased daytime exposure to high-intensity lighting. Traditionally, morning light therapy has been considered a primary approach based on the assumption that poor synchronization of the circadian clock is the leading cause of increased sleep–wake fragmentation. However, several studies suggest that increased sleep–wake fragmentation may result from diminished circadian amplitude rather than poor synchronization. This study aimed to investigate whether the timing of light exposure influences sleep–wake consolidation by analyzing the natural light exposure patterns of older men living in the community. **Materials and Methods:** Using data from the Osteoporotic Fractures in Men study (MrOS), we calculated sleep–wake fragmentation in older men (n = 877) based on one week of wrist actigraphy. Sleep–wake fragmentation was defined as light exposure from actiwatches was quantified in three ways: (1) average white light exposure per minute; (2) mean of sum of total white light exposure; and (3) minutes spent in >1000 lux (time above threshold, TALT). We also examined the relationships between sleep–wake fragmentation, light exposure patterns, and various physical and mental health metrics. **Results**: Our analysis showed that greater sleep–wake fragmentation was linked to poorer physical and mental health, greater impairments in performing instrumental activities of daily life, and worse cognitive functioning. Notably, reduced light exposure during the day was associated with increased sleep–wake fragmentation. Morning and evening light exposure (>1000 lux) did not significantly differentiate between low and high levels of sleep–wake fragmentation. In contrast, increased afternoon light exposure was a better indicator. The most effective differentiation between low and high sleep–wake fragmentation occurred approximately 6.7 h after waking. **Discussion and Conclusions:** These results suggest that a higher degree of sleep–wake fragmentation is indeed associated with worse mental and physical health outcomes. Increased sleep–wake fragmentation was also associated with less daytime light exposure. More importantly, results suggest that light exposure in the early afternoon might be most important for sleep–wake consolidation. During this time of day, light has a notable impact on circadian amplitude while exerting minimal influence on the timing of the circadian clock (Jewett et al., 1994), aligning with the hypothesis that light can induce circadian amplitude changes. Since this study is cross-sectional and correlational, it does not establish causality; nevertheless, the data support the notion that targeting light exposure during this period might benefit older individuals dealing with fragmented sleep. We intend to investigate this hypothesis in more depth through an intervention study.

### 2.63. Technology Use and Evening Activities Affect Sleep Duration and Sleep Quality on Workdays and Work-Free Days—Results from a Survey Study

Thomas Kantermann ^1^, Friederike Mork-Antony ^1^, Ralph Gutknecht ^2^, Kerstin Cuhls^2^

^1^  Institute for Labor and Personnel (IAP), University of Applied Sciences for Economics and Management (FOM), Essen, SynOpus, Bochum, Germany
^2^  Fraunhofer Institute for Systems and Innovation Research (ISI), Karlsruhe, Germany

**Abstract:Background**: The circadian system helps the body anticipate changes in the environment and structure body processes in a timely manner. The circadian system is therefore fundamental to maintaining health, for example, by controlling natural sleep–wake behavior. Deviations in this rhythmic coordination are associated with disorders of sleep, well-being, and health. The central environmental signal for synchronizing the circadian system with the environment (entrainment) is light, which serves as a Zeitgeber for the body. The strongest Zeitgeber for the circadian system is daylight. Current research is, for example, addressing the question of how much daylight is necessary to synchronize the circadian system and to what extent artificial light sources can have an impact on the circadian system. The question arises as to whether there are technologies that prevent people from sleeping and, due to their design, also emit light and in this way can influence or disrupt the circadian system. We investigated these (and more) questions through a research program that included a survey, horizon scanning, and workshops with experts from different but related fields. This presentation shows the results of the survey on sleep, technology use, and evening activities on workdays and non-workdays. **Methods**: In November 2022, we conducted a representative survey among 2000 people aged 18 and older in Germany. Data collection took part within the project CIRCADIA, which was a project funded by the Federal Ministry of Education and Research (BMBF, Germany) as part of the INSIGHT program on interdisciplinary perspectives on social and technological change (Project ID: 16INS106B). In that survey, participants were asked to fill in questions about their everyday life, work, sleep, social practices, and their use of technology. We also asked participants whether they regularly delay their sleep, to finish certain evening activities, despite being aware that this behavior can result in increased levels of tiredness the next day. **Results**: Sleep duration and sleep quality differed between workdays and work-free days. The behavior of delaying sleep to finish certain evening activities was indicated by a substantial number of people. Technology use (e.g., watching TV, smartphone use) and other activities (e.g., chatting with friends/family) in the evening did affect sleep duration and sleep quality. Technology use and evening activities affected sleep duration and sleep quality on both workdays and work-free days. **Conclusions**: Technology use and evening activities are an integral part of everyday life, with the potential to affect sleep duration and sleep quality on workdays and work-free days.

### 2.64. The Acute Effects of Artificial Light on Aspects of Attention and Emotional Processing

Elisabeth Flo-Groeneboom ^1^, Louise Bjerrum ^1^, Oda Bugge Kambestad ^1^, Lin Sørensen ^2^, Berge Osnes ^1^, Malika Elise Hansen ^1^, Endre Visted ^1^, Inger Hilde Nordhus ^1^

^1^  Department of Clinical Psychology, University of Bergen, Bergen, Norway
^2^  Department of Biological and Medical Psychology, University of Bergen, Bergen, Norway

**Abstract:Introduction**: The ENLIGHT study investigates the impact of different light conditions on sustained attention and emotional stimuli processing, research areas that have not been extensively studied. **Methods:** Over four days (09:00–10:30), participants underwent neuropsychological tests under four conditions: blue (ʎ_max_ = 455 nm, melanopic equivalent daylight illuminance (m-EDI) = 1441.8 lx), red (ʎ_max_ = 625 nm, m-EDI = 3.8 lx), bright white (m-EDI = 1155.8 lx), and dim (m-EDI < 10 lx) light, in a randomized, cross-balanced order. Sustained attention was assessed using the psychomotor vigilance task (PVT), and an emotional Go/No-Go task was used to study emotional stimuli processing. Two generalized linear mixed effects models with a number of PVT lapses (response times, RTs ≥ 500 ms) and RT variability (standard deviations in RT/mean RT) as independent variables were conducted controlling for light condition, time, chronotype, season, sex, and consumption of caffeine. For the Go/No Go, three separate two-level (light, time) linear mixed-effects analyses were conducted to investigate the relationship of RT, false alarms (FA), and RTV, controlling for sex and age. **Results**: This study included 40 young adults (24 females, mean age: 21.7, SD = 2.6 years). For the PVT, participants had fewer lapses in blue and bright white light compared with dim light (p’s_corrected_ < 0.05). Participants also had fewer lapses in blue and red compared with dim light, and lower RTV during autumn (p’s_corrected_ < 0.05 and p’s_corrected_ < 0.01). For the emotional Go/No-Go task, more FA errors occurred in dim light compared with blue light (*p* < 0.05). The difference in RTV between the dim condition and blue light condition was significant (*p* < 0.05), as were the differences between red and blue (*p* < 0.01), and white and blue light conditions (*p* < 0.001). Noticeably, both dim and red light showed significant interaction with RTV to happy faces (*p* < 0.001 and *p* < 0.01, respectively). **Conclusions**: High m-EDI light positively influenced lapses on the PVT and reduced FA in the emotional Go/No-Go task. While light did not affect RTV in the PVT, it was lower in autumn than in spring. Conversely, in the Go/No Go, there was an interaction effect between RTV related to happy faces in the dim and red light conditions.

**Keywords**: acute effects; daytime; attention; affect

**Funding:** Research Counsel Norway (275305).

### 2.65. Circadian Mechanisms Related to Vasopressin and Its Associations with Psychiatric Disorders

Sebastian Holst

Roche, Basel, Switzerland

**Abstract:** Vasopressin antagonists have emerged as promising agents in the regulation of circadian rhythms, particularly within the suprachiasmatic nucleus (SCN). This presentation delves into the role of vasopressin antagonists in modulating circadian mechanisms and their potential therapeutic implications for circadian disorders with a special focus on bipolar disorder. This presentation will explore how the vasopressin system facilitates rapid resynchronization of circadian rhythms, in a different manner than traditional Zeitgebers, such as light and melatonin. Additionally, the clinical implications of these findings will be discussed, including the potential for vasopressin antagonists to mitigate circadian disruptions commonly observed in bipolar disorder.

### 2.66. Modification of Light and Dark Transition Length Affects Sleep and Waking Behavior in the Laboratory Mouse

Jeffrey Hubbard

Université de Lausanne, Lausanne, Switzerland

**Abstract:** Sleep and waking in nocturnal rodents are known to be impacted by light exposure through circadian and direct (non-circadian) effects, governed by distinct neuroanatomical structures. However, the fixed light and dark cycles used in many laboratory settings may be suboptimal for these animals, who evolved under natural conditions. Specifically, the presence of fluorescent bulbs that emit multiple peaks across the visible spectrum and the implementation of immediate transitions from light to dark and vice versa, could unduly influence their natural sleep and waking behavior. The modification of this lighting environment, both spectrally through the use of light-emitting diodes (LEDs) and the progressive increase and decrease in the luminance levels, has not yet been fully elucidated. In the current study, we implanted 8 adult (12–24 weeks) male C57Bl/6J mice with electroencephalogram (EEG) and electromyogram (EMG) electrodes to record sleep and waking behavior and exposed them to a custom-built LED-driven light-dark environment with several dark-to-light and light-to-dark transition paradigms for seven days each for 15 min, 30 min, 45 min, and 60 min, in addition to a comparison with standard fluorescent tubes (0 min). Each lighting condition was followed by 7 days of standard 12:12 light-dark cycles (0 min transition). Sleep and waking states were then quantified using standard criteria and qualitative analysis of the signals was also performed. Furthermore, locomotor activity was captured using single-pass infrared movement detectors to verify intact circadian rhythms. A wide array of quantitative sleep–wake parameters were affected by the modification of the transitions periods, including time spent in wake, non-rapid-eye movement (NREM) sleep, and rapid-eye-movement (REM) sleep, as well as bout length distribution. In addition, spectral analysis identified differences between the transitions not only during light periods but also subsequent dark periods. Interestingly, mice also began anticipating these modified transition schedules after initial exposure. Finally, a comparison of the immediate (0 min) regime between fluorescent tubes and LEDs yielded different results across several sleep–wake parameters. These results demonstrate that prolonging the transition from light-to-dark and dark-to-light, as well as using LEDs, significantly impacts both the quality and quantity of sleep–wake behavior. Furthermore, these data underscore a need to reevaluate how the controlled environment of a laboratory animal may affect a wide variety of behaviors and physiology and may need to be altered to mimic more natural conditions. This work was funded by the Swiss National Science Foundation and the University of Lausanne.

### 2.67. Translating Circadian Science to the Clinic

Sabra Abbott

Northwestern University, Evanston, IL, USA

**Abstract:** Circadian dysfunction can have a negative impact on a wide range of disease processes. In the Circadian Medicine clinic, our goal is to develop better tools to diagnosis and manage individuals with circadian dysfunction. As one example, in population-based studies, individuals with later chronotypes tend to experience more negative health outcomes than those with intermediate or early chronotypes. Within the group of individuals who attended to the clinic for management of delayed sleep–wake timing, the underlying pathophysiology appeared to be quite heterogeneous, and thus it was not surprising that available treatment options had mixed effectiveness. Our work has identified a population of individuals with severe delayed sleep–wake phase disorders and sighted non-24 h sleep–wake rhythm disorders who appear to have impaired light sensitivity. Further efforts are now focused on developing better strategies to identify these individuals within the clinic population, and implementing novel treatment approaches based on these findings.

### 2.68. Circadian Dysregulation in Cardiovascular Disease and Circadian Medicine Treatments

Tami Martino

University of Guelph, Guelph, ON, Canada

**Abstract:Background**: The circadian clock is essential for all life on earth, regulating daily rhythms in human biology. This is especially relevant for a healthy cardiovascular system, influencing daily rhythmicity in heart rate, blood pressure, and endocrine hormones. Circadian rhythms also underlie the timing of the onset of adverse cardiovascular events, such as heart attacks (myocardial infarction, MI), high blood pressure (hypertension), sudden cardiac death, and surgical outcomes based on the time of day. **Methods and Results**: Our early research investigates the 24 h molecular rhythms that underlie the day/night physiology of the cardiovascular system. We have studied how maintaining normal daily rhythms and how disrupting circadian rhythmicity contributes to cardiovascular disease. For our studies, we use a variety of genetic murine models, environmental desynchrony, shift work models, chronopharmacology, and molecular “omics” approaches. Our recent focus is on applying circadian biology to clinical medicine for treating cardiovascular disease. Approaches include the following: (1) Chronotherapy-timing treatments to enhance efficacy. (2) Circadian lighting to optimize light exposure for better intensive care. (3) Chronopharmacology to use small molecule modulators to target the circadian mechanism, particularly for benefitting cardiac repair post-myocardial infarction and preventing heart failure. We are also establishing a preclinical translation center to advance promising preclinical therapies from bench to bedside. This presentation will cover our published and recent research findings and their implications for cardiovascular disease and recovery. **Conclusions**: Circadian rhythms are vital for many bodily functions. Our research highlights their importance in healing from disease or injury, leading to circadian medicine treatments for cardiovascular disease.

**Keywords**: circadian medicine; cardiovascular; chronotherapy; dysregulation.

**Funding**: Canadian Institutes for Health Research (CIHR) and Heart and Stroke Foundation of Canada (HSFC).

### 2.69. Shift Work, Circadian Disruption and Sleep Health

Shantha Rajaratnam

Monash University, Melbourne, Victoria, Australia

**Abstract:** Shift work is reported to account for between 15 and 20 percent of the workforce in many countries and is likely to increase in frequency as flexible work arrangements become more common, the gig economy expands, and globalization of the workforces increases. Shift work is widely reported to increase the risk of several adverse health conditions including sleep disorders, obesity, cardiovascular disease, diabetes, cancer, reproductive disorders, gastrointestinal disturbances, and mental health conditions such as depression, anxiety, stress, and burnout. This presentation will examine circadian misalignment and sleep loss in a range of shift working industries, in particular healthcare workers, and associations between shift work and adverse health, safety, and performance outcomes. Current and emerging approaches to manage the health, safety, and productivity impacts associated with shift work will be discussed. These include rostering/scheduling programs and a digital personalized sleep–wake application. https://www.workalert.org.au/. The urgent need for standardization in clinical management of shift work disorder and other circadian rhythm sleep–wake disorders, and for translation of research into practice, has led to the establishment of the International Association of Circadian Health Clinics. The new association will be discussed.

### 2.70. Clinical Implications of Spectral Differences in Phototherapy Devices

Jana Kopřivová ^1^, Lenka Maierova ^2^, Kateřina Červená ^3^, Zdeňka Bendová ^4^

^1^  National Institute of Mental Health, Prague, Czech Republic
^2^  Czech Technical University in Prague, Prague, Czech Republic
^3^  Department of Molecular Biology, Umeå University, Umeå, Czech Republic
^4^  Faculty of Science, Charles University, Prague, Czech Republic

**Abstract:Background**: Modern LED technology has created sources of white light with different spectral compositions and thus different biological efficacies, high surface luminance, and high energy efficiencies. Technology also allows manufacturers to tailor the spectral composition to the needs of the application, for example, to maximize the energy of a selected spectral region. The growing understanding of the non-image forming (NIF) effects of light underlines the importance of spectral composition. For more than 40 years, phototherapy has been used to effectively support mental health, treating SAD and other affective disorders. Treatment guidelines recommend a (photopic) illuminance of 10 000 lx at the patient’s eye. These guidelines, originally based on fluorescent light sources, remain in use with minor international variations and do not consider spectral composition. **Methods**: To detect differences and uncertainty associated with underestimating the importance of spectra in phototherapy, we decided to assess NIF effects of different devices. We collected data from traditional fluorescent and modern LED phototherapy devices, measuring photopic illuminance at the eye, spectral composition, and luminance of the light-emitting surface. Melanopic effects were calculated according to the international standard CIE S 026/E:2018. We compared the obtained melanopic ELR, DER, and EDI between devices, and recommended upper and lower limits for melanopic EDI based on the original guidelines. These limits were verified in studies of healthy subjects and patients using a high melanopic-inducing LED light source. **Results**: Comparison of phototherapy devices with different technologies showed significant differences in calculated melanopic irradiance, which varied up to twofold for white light-emitting devices and up to tenfold for monochromatic (blue light) devices. The melanopic irradiance of the reference device with fluorescent technology was 0.989 mW.lm^−1^. The recommended melanopic EDI was set between 7500 and 1800 lx. The tests showed, among other things, that exposure to light at both a melanopic EDI of 7500 and 2100 lx reduced negative affect and altered EEG activity in brain regions (especially the insula) involved in emotional processing. However, regular two-week exposure to melanopic EDI 10 300 lx led to an increase in the sleep fragmentation index, whereas regular two-week exposure to melanopic EDI 2300 lx had no effect on sleep fragmentation. **Conclusions**: Existing recommendations for phototherapy do not consider the effects of light spectrum. They should be extended to include the NIF effect of light and the capabilities of new LED technology. Developers should provide melanopic ELR, DER, or EDI information, which should replace photopic illuminance in treatment protocols. This change would allow comparison of devices and treatment continuity when switching technologies and allow different devices (stationary, portable, with varying therapeutic effects) to be tailored to individual patient needs. In addition, attention must be paid to the maximum luminance of the light-emitting surface to prevent exposure to excessive harmful blue light.

**Keywords**: NIF; bright light therapy; phototherapy; LED; melanopic; guidelines

**Funding:** The project “Stationary and mobile devices to support circadian synchronization, treatment and prevention of mental disorders through full spectrum-light phototherapy”, FW02020025, has been funded by the Technology Agency of the Czech Republic and the Ministry of Industry and Trade of the Czech Republic within the TREND Programme.

